# Multi-Omic Data Integration Allows Baseline Immune Signatures to Predict Hepatitis B Vaccine Response in a Small Cohort

**DOI:** 10.3389/fimmu.2020.578801

**Published:** 2020-11-30

**Authors:** Casey P. Shannon, Travis M. Blimkie, Rym Ben-Othman, Nicole Gladish, Nelly Amenyogbe, Sibyl Drissler, Rachel D. Edgar, Queenie Chan, Mel Krajden, Leonard J. Foster, Michael S. Kobor, William W. Mohn, Ryan R. Brinkman, Kim-Anh Le Cao, Richard H. Scheuermann, Scott J. Tebbutt, Robert E.W. Hancock, Wayne C. Koff, Tobias R. Kollmann, Manish Sadarangani, Amy Huei-Yi Lee

**Affiliations:** ^1^ Prevention of Organ Failure (PROOF) Centre of Excellence and Centre for Heart Lung Innovation, St. Paul’s Hospital, Vancouver, BC, Canada; ^2^ UBC Centre for Heart Lung Innovation, St. Paul’s Hospital, Vancouver, BC, Canada; ^3^ Department of Microbiology and Immunology, Life Sciences Institute, University of British Columbia, Vancouver, BC, Canada; ^4^ Department of Pediatrics, University of British Columbia, Vancouver, BC, Canada; ^5^ Telethon Kids Institute, Perth Children’s Hospital, University of Western Australia, Nedlands, WA, Australia; ^6^ Centre for Molecular Medicine and Therapeutics, BC Children’s Hospital Research Institute, Department of Medical Genetics, The University of British Columbia, Vancouver, BC, Canada; ^7^ Department of Experimental Medicine, University of British Columbia, Vancouver, BC, Canada; ^8^ Terry Fox Laboratory, British Columbia Cancer Agency, Vancouver, BC, Canada; ^9^ European Molecular Biology Laboratory, European Bioinformatics Institute, Wellcome Genome Campus, Cambridge, United Kingdom; ^10^ Department of Biochemistry & Molecular Biology, Michael Smith Laboratories, University of British Columbia, Vancouver, BC, Canada; ^11^ British Columbia Centre for Disease Control, Vancouver, BC, Canada; ^12^ Melbourne Integrative Genomics, School of Mathematics and Statistics, The University of Melbourne, Parkville, VIC, Australia; ^13^ Department of Informatics, J. Craig Venter Institute, La Jolla, CA, United States; ^14^ Department of Pathology, University of California, San Diego, CA, United States; ^15^ Division of Vaccine Discovery, La Jolla Institute for Immunology, La Jolla, CA, United States; ^16^ Department of Medicine, Division of Respiratory Medicine, University of British Columbia, Vancouver, BC, Canada; ^17^ Human Vaccines Project, New York, NY, United States; ^18^ Vaccine Evaluation Center, BC Children’s Hospital Research Institute, Vancouver, BC, Canada; ^19^ Department of Molecular Biology and Biochemistry, Simon Fraser University, Burnaby, BC, Canada

**Keywords:** multi-omic analysis, hepatitis B vaccination, baseline immunity, network analysis, vaccine response

## Abstract

**Background:**

Vaccination remains one of the most effective means of reducing the burden of infectious diseases globally. Improving our understanding of the molecular basis for effective vaccine response is of paramount importance if we are to ensure the success of future vaccine development efforts.

**Methods:**

We applied cutting edge multi-omics approaches to extensively characterize temporal molecular responses following vaccination with hepatitis B virus (HBV) vaccine. Data were integrated across cellular, epigenomic, transcriptomic, proteomic, and fecal microbiome profiles, and correlated to final HBV antibody titres.

**Results:**

Using both an unsupervised molecular-interaction network integration method (NetworkAnalyst) and a data-driven integration approach (DIABLO), we uncovered baseline molecular patterns and pathways associated with more effective vaccine responses to HBV. Biological associations were unravelled, with signalling pathways such as JAK-STAT and interleukin signalling, Toll-like receptor cascades, interferon signalling, and Th17 cell differentiation emerging as important pre-vaccination modulators of response.

**Conclusion:**

This study provides further evidence that baseline cellular and molecular characteristics of an individual’s immune system influence vaccine responses, and highlights the utility of integrating information across many parallel molecular datasets.

## Introduction

Hepatitis B is a viral infection that primarily affects the liver of infected individuals, and can cause both acute and chronic disease. The WHO estimates that 257 million people had a chronic hepatitis B infection in 2015 (1), with nearly one million deaths occurring as a result of hepatitis B infections causing cirrhosis and liver cancer. Fortunately, there are highly efficacious vaccines available for hepatitis B with rates of protection above 90% if given in a two- or three-dose schedule (2). The antibody response to hepatitis B virus (HBV) vaccine is one of the best correlates of protection from infection with well characterized quantitative levels associated with degree of protection, allowing clinicians and researchers to easily determine if an individual is sufficiently protected after receipt of the vaccination series.

Unfortunately, the response to vaccination is highly variable in older adults, with some individuals quickly producing high levels of HBV antibodies, while others never develop protective levels (3). While this can be overcome by additional booster doses, the reasons for this reduced efficacy in older populations remains unclear. Age-related immuno-senescence is one proposed mechanism, but a better understanding of the reasons that underlie this variable response in older adults is still needed. This could be accomplished with a large study involving many individuals, but recruiting large numbers of participants for vaccine studies can be difficult and costly. Researchers are thus tasked with attempting to draw significant and meaningful conclusions from relatively small cohorts, typically assessed using only a small variety of methods. To overcome this issue, we used multiple omics technologies together with computational integration methods to generate a more comprehensive picture of vaccine response.

Here, in a cohort of 15 healthy adults ranging from 44 to 73 years of age, we profiled a broad variety of molecular modalities from peripheral whole blood, including immune cell composition, DNA methylation, gene expression, protein abundance, as well as fecal 16S microbiome, to provide the most comprehensive picture of the immune response to an aluminium-adjuvanted HBV vaccine. Antibody measurements to HBV surface antigens were used as the quantitatively defined endpoint in our model to address two main questions: (1) can we identify baseline immune signatures that predict vaccine responses and differentiate between responders and non-responders, and (2) what temporal molecular changes occur following HBV vaccination? Baseline differences correlating with final HBV vaccine response could be identified in this small (n = 15) cohort of adults by using a multi-omics integration approach. This general concept of specific baseline immune signatures predicting vaccine responses has been demonstrated in large cohort studies in the context of HBV, influenza, and malaria vaccines (4–7). However, the benefit of integrating multi-omics baseline data in the context of small sample size has not previously been documented. This approach has substantial implications not only in the field of bioinformatics-driven analyses, but also in systems vaccinology and vaccine development.

## Materials and Methods

### Participant Recruitment and Study Design

**Figure 1:**
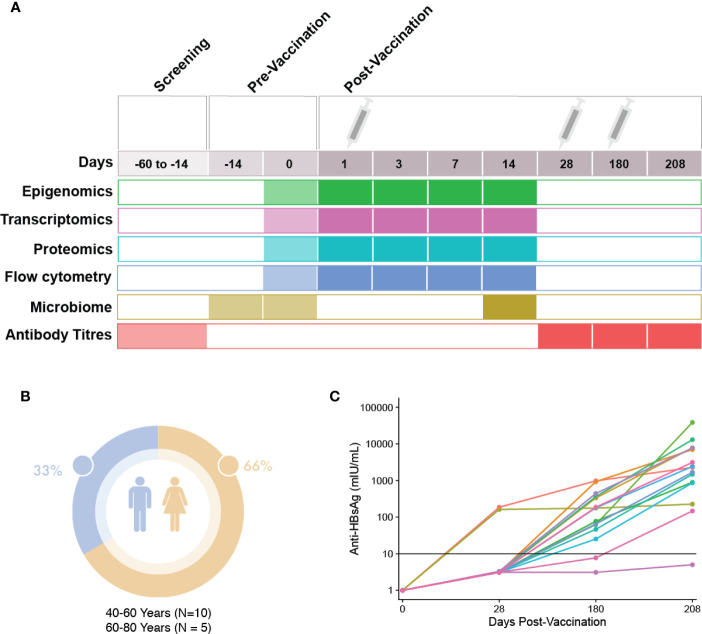
Study visit schedule and cohort demographics. **(A)** Immunization and sampling schedule: Screening of patients eligible for this study occurred 14–60 days prior to the first vaccine dose. Eligible participants returned 14 days prior to vaccination to complete enrolment and have blood and microbiome samples taken. At day 0, the first vaccine dose was administered after blood and microbiome sampling. Blood sampling then occurred at days 1, 3, 7, and 14 post-vaccination. At day 28, blood sampling and the second HBV dose was administered. At day 180, blood sampling and the last dose of HBV was given, followed by a final blood sample taken at day 208. **(B)** Demographics: Participant sex and age. **(C)** Patient anti-HBs antibody titres at 28, 180, and 208 days post first HBV vaccination dose.

A prospective, observational study (ClinicalTrials.gov; NCT03083158) of immune responses to the HBV vaccine (ENGERIX^®^-B) was undertaken, with recruitment occurring at the Vaccine Evaluation Center (VEC), BC Children’s Hospital Research Institute in Vancouver, Canada. Participants were recruited by e-mail, mail and telephone. All participants enrolled in the study provided written informed consent under a research protocol (H17-00175) approved by the University of British Columbia Women’s and Children’s research ethics board. All initial sample processing was undertaken at the VEC laboratory. Participants were healthy adults aged 44–73 years who were seronegative to HBV and with no prior history to HBV infection or vaccination, with demographics shown in **Figure 1**. In brief, screening of participants was performed by blood sampling to determine their antibody titers to HBV surface antigens. Participants with anti-hepatitis B surface antigen (HBs) antibody levels under 3.1IU/L were considered seronegative and a total of 15 eligible individuals enrolled to participate in the study. For detailed inclusion/exclusion criteria, see the HBV vaccine Methods manuscript: *Systems biology methods applied to blood and tissue for a comprehensive analysis of immune response to Hepatitis B vaccine in adults* (8). Enrolled individuals attended the first study visit involving the collection of clinical history, a physical examination as well as pre-vaccination biospecimen collection (blood and fecal microbiome samples). One ml (20 micrograms) of HBV vaccine was administered *via* intramuscular deltoid injection at three different times throughout the study (0, 28, and 180 days). At each visit, a number of molecular and clinical tests were performed on the collected biospecimens (**Figure 1**). HBV serology of study participants at baseline were performed at the BC Centre for Disease Control. In total, participants were monitored during 12 visits spanning the course of seven months (8). HBV titres were measured once during the screening phase, and at three additional time points, corresponding to 28, 180, and 208 days after the first dose of HBV vaccine.

### Cellular Profiling, Omics, and Statistical Analysis of the Time Course

Various omics studies were performed as described in the **Supplementary Materials and Methods**, with workflow figure shown in **Supplementary Figure 1**. Briefly, peripheral whole blood cells were profiled by flow cytometry, genome-wide DNA methylation (Illumina Infinium MethylationEPIC BeadChip), transcript abundance (whole blood, bulk RNA-Seq), and proteome-wide protein abundance (mass spectrometry) at various time points (**Figure 1**). Additionally, the bacterial composition (microbiome) of the gut was assessed by 16S rRNA microbiome profiling pre- (Day -14 and 0) and post-vaccination (Day 14). The gating strategy used for immune cell phenotyping is included in **Supplementary Figure S3**.

To identify global changes pre- versus post- vaccination across different omics data, we used multi-level principal component analysis (multi-level PCA) from *mixOmics* to highlight the effect of vaccination (treatment effect) within subjects separately from the biological variation that existed between subjects (9, 10). Based on the temporal trends observed, Day 1 and 14 post-vaccination were further investigated using univariate statistical tests within each omics method to identify differentially methylated CpG sites, expressed genes, and proteins following vaccination (refer to **Supplementary Materials and Methods** for further details on each of these methods).

### Identifying Features Associated With HBV Vaccine Response From Single Omics Data

To identify baseline differences between participants who responded to vaccine and those who did not, we used the HBV-specific antibody titre levels from Day 180 to divide the participants into either responders or non-responders, based on the well-established correlate of protection of 10 mIU/ml (6). This demarcation was used in analyzing the transcriptomic and proteomic data. For analysis of the epigenetic data, the same titre values from Day 180 were instead treated as a continuous variable. For more details on how each of these datasets were analyzed, refer to the **Supplementary Methods** section.

Lists of genes or proteins identified through these methods were submitted to NetworkAnalyst (11, 12) for unsupervised construction of Protein-Protein Interaction (PPI; direct, metabolic or regulatory interactions) networks, to facilitate biological enrichment of the results. In these PPI networks, nodes represent individual proteins, while the edges which connect nodes correspond to a known, curated interaction between a given pair of proteins. Node tables representing all members of a network were downloaded from NetworkAnalyst to test for enriched Reactome pathways using the R package *Sigora* (13), with pathways being considered significantly enriched with a Bonferroni-corrected p-value of <0.001.

### Identifying Features Associated With HBV Vaccine Response From Multi-Omic Data

To identify features that could be used to predict vaccine response (anti-HB titres) from baseline omics profiles, we used two complementary data integration strategies: NetworkAnalyst and DIABLO.

#### NetworkAnalyst

NetworkAnalyst is an online tool which leverages known protein-protein interactions to construct biological networks in an unsupervised manner to provide biological insights (11, 12). Genes or proteins identified when comparing responders and non-responders (using Day 180 titres as detailed previously) using combinations of three different omics data (epigenetics, proteomics, and transcriptomics) were uploaded to NetworkAnalyst and combined to build minimally-connected first order PPI networks, with the commonly-occurring promiscuous node UBC (Ubiquitin C; 10,837 known interactions at www.innatedb.com) removed. To highlight novel nodes in the combined networks, networks were constructed individually for the different omics methods and their node tables downloaded to enable comparison to the node table from the combined network. This allowed identification of nodes that were present in the combined network, but absent when examining each omics network separately. Node tables downloaded from NetworkAnalyst were tested for enriched Reactome or KEGG pathways using the R package *Sigora* as previously described (13, 14).

#### DIABLO

DIABLO, part of the *mixOmics* framework, is a supervised, data-driven, hypothesis-free multi-omics integration approach that has been successfully applied, by us and others, to derive novel, robust biomarkers, and increase our understanding of the molecular regulatory mechanisms that underlie health and disease (15–17). DIABLO extends sparse Generalized Canonical Correlation Analysis (sGCCA) for multi-omics and supervised integration (18, 19). DIABLO performs multivariate dimensionality reduction and selects correlated variables across different datasets by maximizing the covariance between linear combinations of variables (latent component scores), across datasets (blocks; flow cytometry, epigenomic, transcriptomic, and proteomic profiling, fecal 16S rRNA microbiome) and an outcome variable (response; log-transformed anti-HBs IgG level measured at the final follow-up). Feature selection is performed internally using lasso penalties. The data are then projected into a smaller dimensional subspace spanned by the components for prediction. The ability of the integrative model to predict final anti-HBs IgG titres was then evaluated using leave-one-out cross-validation.

### Mapping of Identifiers to Facilitate Biological Interpretation

To facilitate biological interpretation, features were mapped, where possible, to HUGO Gene Nomenclature Committee (HGNC) gene symbols. Methylated CpG dimers were mapped using the annotation provided by Illumina (*IlluminaHumanMethylationEPICanno.ilm10b2.hg19* R package). Ensemble gene IDs and UniProt protein IDs were mapped using the Biomart service from Ensembl (20). Gene set enrichment was assessed against the Broad Institute’s MSigDB (C2 collection: manually curated gene sets from KEGG, REACTOME, etc.) using a hypergeometric test, or *Sigora*, as detailed previously (13).

## Results

### Response of Older Adults to a Three-Dose Schedule of HBV Vaccine

To enable analyses aimed at identifying differences between HBV vaccine responders and non-responders, we first examined the titre levels for each participant over the course of this study. As described previously, participants’ anti-HB titres were measured three times following the first dose of HBV vaccine (**Figure 1C**). At the first antibody titre measurement on Day 28, after only a single dose of HBV vaccine, 2 out of 15 participants (aged 63 and 72, both female) showed titres that would classify them as responders, with titre levels above the correlate of protection, 10 mIU/ml. Based on a multi-level PCA, we saw little difference between these two individuals and the remainder of our cohort (**Supplementary Figure S5**). By Day 180, after having received two doses, 13 of 15 participants showed titre levels equal to or greater than 10mIU/mL, measures which have been shown to correlate well with protection. At the final titre measurement 30 days after the third vaccination (208 Days after the first dose), all but a single participant showed titre levels above the correlate of protection. We also examined if there was any relationship between DNAm-based age acceleration and titre levels at Day 180, and found no correlation (**Supplementary Figure 4**).

### Immune Cell Phenotyping

To identify potential immune cell types important for HBV vaccine responses, Spearman correlation analysis was performed using the baseline counts of various immune cell types (defined by 15 anchor makers; **Supplementary Table 1**) and the HBV antibody titres measured at Day 180. No statistically significant baseline cell type differences were identified from correlations to Day 180 titres (**Supplementary Figure 2**). However, we observed a trend of positive correlation between CD3^+^ T cells on Day 7 and 14 to HB antibody titres measured at Day 180. In contrast, we observed a trend of negative correlation between CD56^dim^ CD16^+/-^ NK cell populations on Day 7 and 14 to HB antibody titres measured at Day 180. While there was no definitive immune phenotype that could potentially identify vaccine responders to non-responders, our data suggested that T cell subsets might potentially be important in the immune response to hepatitis B during infection or vaccination (21).

### Molecular Changes Following Vaccination

**Figure 2:**
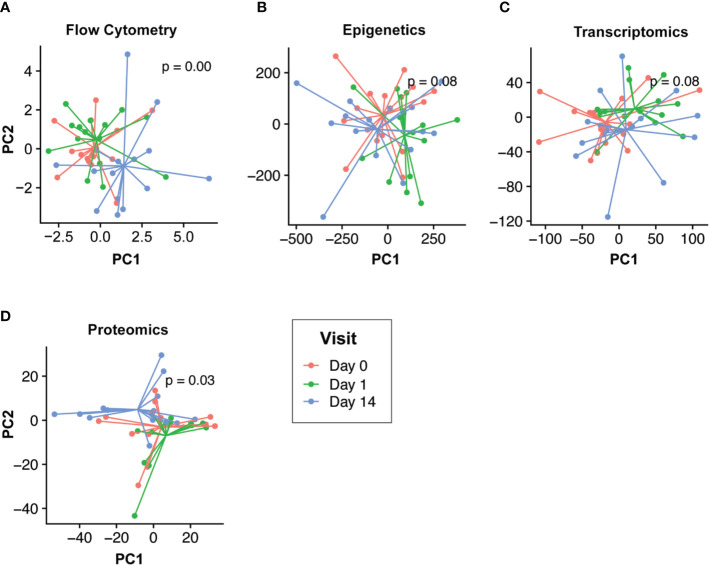
Temporal response profiles following HBV vaccination differs across omics compartments. Low dimensional projection of the flow cytometry **(A)**, epigenomic **(B)**, transcriptomic **(C)**, and proteomic **(D)** data using multilevel principal component analysis to visualize global changes across time. In each panel, different post-vaccination time points for each individual are shown in red (Day 0), green (Day 1), and blue (Day 14). We observed differing global temporal patterns of change following vaccination across the various omics compartments. Epigenomic and transcriptomic profiles changed rapidly post-vaccination (Day 1; green vs. blue/red) before returning to baseline by Day 14. Conversely, flow cytometry and proteomic profiles were most distinct by Day 14 (blue vs. red/green).

Our goal was to first define the molecular changes that occurred following HBV vaccination. To do this while removing intra-individual differences, we performed multi-level PCA of the flow cytometry, epigenomic, transcriptomics, and proteomic data (**Figure 2**). For both the epigenomic and transcriptomic profiles, we observed rapid changes one day after HBV vaccination, followed by a return to baseline on Day 14 (**Figures 2B, C**). In contrast, cell population and proteomic profiles were most distinct from baseline two weeks after HBV vaccination (**Figures 2A, D**).

**Figure 3:**
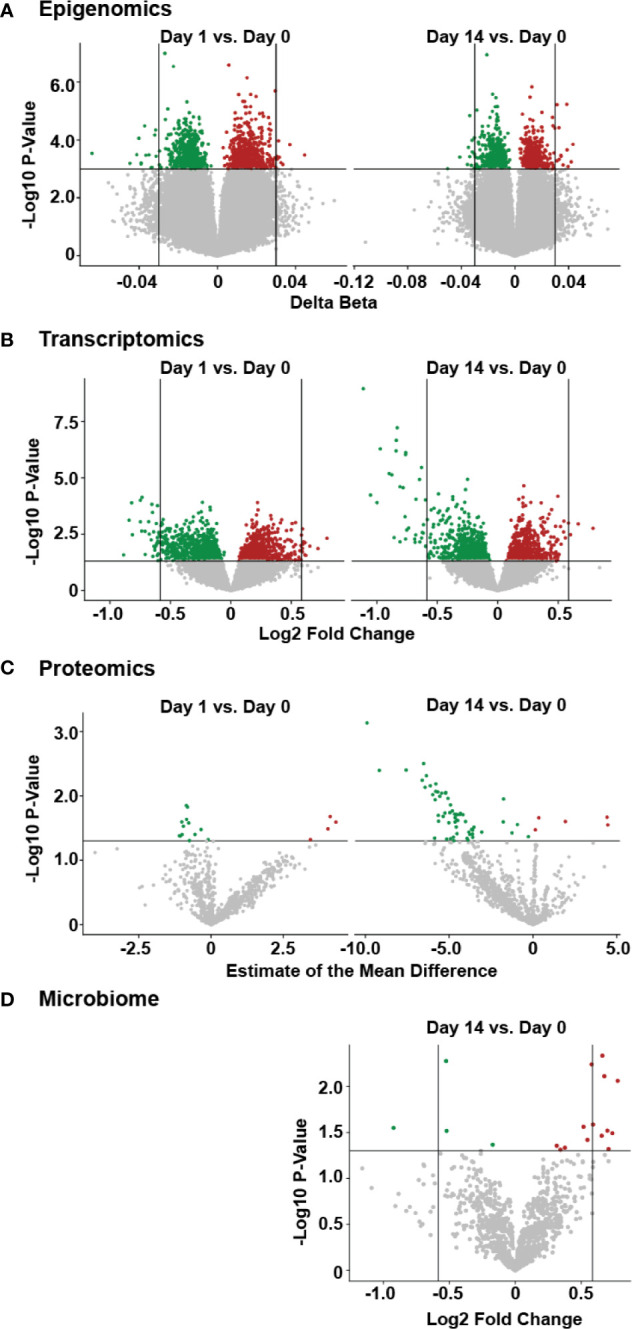
Temporal molecular changes identified in four omic data following HBV vaccination. **(A)** Volcano plot of epigenetic data, with horizontal lines indicating a p-value of 0.001, and vertical lines indicating a delta beta of 0.03. **(B)** Volcano plot for transcriptomic data, with horizontal line indicating a p-value of 0.05 and vertical lines indicating fold change of 1.5. **(C)** volcano plot of proteomic data, with horizontal line indicating a p-value of 0.05. **(D)** volcano plot of microbiome data, with the horizontal line indicating a p-value of 0.05 and vertical lines indicating a fold change of 1.5, with larger fold changes to the left and right of these lines. For all panels, green or red points indicate a decrease or increase, respectively, in the post-vaccine sample compared to the pre-vaccine baseline. Higher values on the y-axis for all plots indicate greater significance (lower p-value).

From the epigenomic data, we identified a total of 18 unique DNA methylation sites using a univariate analysis, located in twelve genes that were significantly differently methylated following vaccination, when compared to baseline (**Figure 3A**, **Supplementary Table 2**). A number of these genes are known to participate in immune functions, including: BAIAP2L1 that plays a role in actin organization; a cytotoxic and regulatory T Cell-associated molecule (CRTAM); a negative regulator of TGF-β signalling LDL receptor (LDLRAD4); a transcriptional repressor of activation protein-1 (ZNF12); anti-viral and cytidine deaminase (APOBEC3A_B); and a guanine exchange factor and endosome dynamics regulator (ANKRD27). Similarly, we observed minimal transcriptomic changes following HBV vaccination, with only 14 significantly differentially expressed (DE) genes (adjusted p-value <0.05 and absolute fold change >1.5; **Supplementary Table 3**) when comparing Day 14 to pre-vaccination Day 0 (**Figure 3B**). Among these were the genes CAMP (22), encoding host defence peptide LL-37 that has a known association with immune and inflammatory responses, and the neutrophil-associated elastase gene ELANE (23), which can alter the roles of NK cells, monocytes, and granulocytes. These results point to a detectable change in the immune response of inoculated individuals as early as two weeks after having received the vaccine. No statistically significant changes were observed in proteomics or the fecal microbiome following vaccination (**Figures 3C, D**).

### Influence of Immune Baseline on Vaccine Response

**Figure 4:**
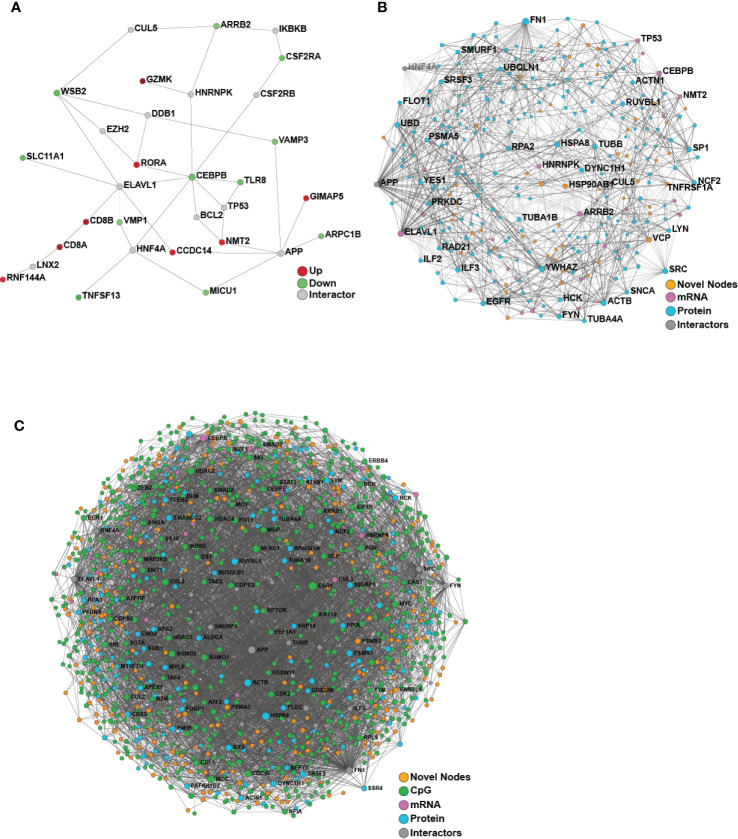
Network analysis of transcriptomics and proteomics data reveal baseline differences between vaccine responders and non-responders. **(A)** Minimum-connected network from the 40 DE genes identified when comparing responders to non-responders (defined using Day 180 titre measures). **(B)** Minimally-connected first-order integrated protein-protein interaction network of the same 40 DE genes combined with the 267 differentially expressed proteins when comparing responders to non-responders (Day 180 post-vaccination). **(C)** Minimally-connected first-order integrated protein-protein interaction network of differentially expressed transcripts and proteins from B with the addition of differentially methylated genes (898 CpG sites) when comparing responders to non-responders (Day 180 post-vaccination). Novel nodes, not present in individual transcriptomic or proteomics networks are highlighted in orange.

We then turned our attention to identifying baseline differences between participants who responded to the HBV vaccine and those who did not based on the well-established correlate of protection of 10 mIU/ml. Comparing the responders and non-responders (defined at Day 180) using only the pre-vaccine transcriptomic data, 40 differentially expressed (DE) genes were identified, and used to construct a minimally-connected first-order PPI network (a first order network in which the interconnecting grey nodes that connect to only a single DE gene are removed), as shown in **Figure 4A**. Some of the genes found to be differentially expressed (adjusted p-value <0.05; **Supplementary Table 4**) included up-regulation of CD8A and CD8B that are involved in cytotoxic T-cell mediated immune responses, THEMIS, implicated in T-cell lineage selection and maturation, and transcription factor RORA that regulates cytokine expression in T-regulatory cells (24, 25). Downregulated genes included CEBPB that acts in the suppression of T-cells through transcription factor MYC and SLC11A1, a divalent metal ion transporter important for iron metabolism and host resistance to pathogens (26).

For the proteomic analysis, we were able to identify 267 unique peptides that changed in expression when comparing responders and non-responders at the pre-vaccine baseline (adjusted p-value <0.05; **Supplementary Table 5**). Some of the proteins identified by this analysis include: monocyte marker CD14, calcium binding inhibitor of HCV replication S100A6 (27), and TRIM25, a mediator of signal transduction in response to viral infections (28, 29). Pathway enrichment with Sigora (Bonferroni-corrected p-value <0.001; **Supplementary Table 6**) yielded multiple pathways, including “Neutrophil degranulation” and “Gene and protein expression by JAK-STAT signalling after Interleukin-12 stimulation”. The greatest number of changes were observed in the epigenomic analysis, with identification of 898 DNA methylation CpG sites located within 632 genes (p-value ≤0.005 and change in beta >5%, with beta defined as proportion of methylated DNA at a particular locus; **Supplementary Table 7**). These genes were enriched for ERBB4 signalling pathways, a tyrosine protein kinase involved in downstream signalling of the B Cell Receptor, Notch-HLH transcription pathways, and implicated in various inflammatory diseases (p-value ≤ 0.005) (full list in **Supplementary Table 8**) (30).

### Multi-Omics Data Integration by a Functional Approach, NetworkAnalyst, Identified Novel Pathways Contributing to Vaccine Responses

Since we were only able to identify limited baseline molecular differences between responders and non-responders from the individual omics data, we next applied a proven (14) multi-omics integration method to identify consistent signatures associated with robust vaccine responses. To determine the molecular and immunological differences that might influence vaccine responses, an integrative analysis was performed using either two or three omics datasets (transcriptomics, proteomics and/or epigenomics) comparing responders vs. non-responders using NetworkAnalyst (**Figure 4**). Both integrations revealed a dense minimally-connected network containing many novel nodes (**Figures 4B, C**, highlighted in orange), including: EOMES (eomesodermin), which is involved in the differentiation of CD8^+^ T cells, active against viral infections (31); VCP involved in T cell activation (32); and EGR1 that stimulates T cell activation and promotes IL2 production (33). Interestingly the T-cell modulatory genes found using transcriptomics were well integrated into this network and several new T-cell modulators were identified, including ILF2 that mediates expression of IL2 by T-cells, PP1A that modulates T-cell cytokine expression, and FN1 which is Th1-specific in humans (34).

To gain mechanistic and biological insight into the immune pathways, we then tested the nodes from **Figures 4B or C** for enriched pathways with *Sigora*, using both Reactome and KEGG databases. Some of the significant pathways include innate immunity pathways such as “Neutrophil degranulation”, “Gene and protein expression by JAK-STAT signaling after Interleukin-12 stimulation”, and “Toll-like Receptor 4 (TLR4) Cascade”. In addition, we identified some signatures of adaptive immune responses such as “IL17 signaling” and “Th17 cell differentiation”, providing further insights into immune differences between responders and non-responders. In particular, JAK-STAT is a major anti-viral pathway that when activated can lead to inhibition of HBV infections (35), while TLR4 activation suppresses HBV infections (36). The full list of enriched pathways is included in **Supplementary Tables 9**–**12**.

### Multi-Omics Data Integration Using a Data-Driven Approach Improved Our Understanding of Vaccine Response in a Small Cohort

In addition, we used the supervised, data-driven, multivariate integration method DIABLO to identify baseline (pre-vaccination) predictors of vaccine responses based on multiple high-throughput datasets (flow cytometry, epigenomic, transcriptomic, and proteomic profiling, as well as fecal 16S rRNA microbiome profiling). To determine whether integrating the data in this manner resulted in models with better predictive performance, we fit DIABLO models of varying complexity (total number of variables selected), and compared them to sparse partial least squares regression [sPLS (18)] models fit on each of the individual high-throughput datasets with similar number of variables selected. We assessed predictive performance using leave-one-out cross-validation and found that the integrative DIABLO model generally outperformed single-omic sPLS models (**Supplementary Figure 6**).

**Figure 5:**
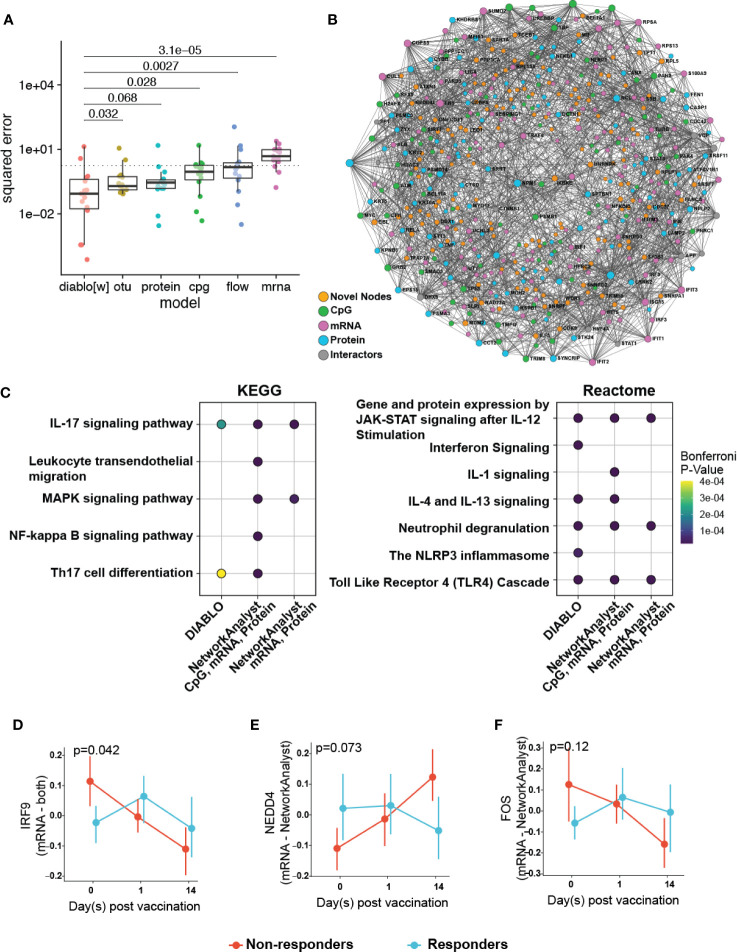
Multi-omics integration to reduce overfitting by identifying of more biologically relevant features. **(A)** Comparison of the performance of a multi-omics model (DIABLO) to that of single-omics models of equivalent complexities, fit separately to individual omics datasets (otu, operating taxonomic units of the fecal microbiome; cpg, blood-based DNA methylation; protein, plasma proteomics; flow, cell counts by flow cytometry; mrna, whole blood transcriptomics). Mean squared error (MSE) differed significantly across all models (Kruskal-Wallis test; p = 0.0023), with the multi-omics model achieving significantly lower error (and better performance) when compared to all other models, with the exception of the proteomics-derived model (p = 0.068). **(B)** Integrated minimally-connected first-order network of features identified by DIABLO from transcriptomic, proteomics, and epigenetic data. Novel nodes identified from integration are highlighted in orange. **(C)** Selected enriched pathways and **(D–F)** selected enriched genes (mRNA) or proteins (proteomics) identified from integration (NetworkAnalyst, DIABLO, or both methods) are shown.

Based on this rigorous statistical assessment, we chose to characterize the variables selected by the DIABLO (17) model that achieved the best overall performance (lowest error rate; **Figure 5A**). Where possible (CpGs, transcripts, proteins) individual features were mapped to gene symbols, while features identified by either the integrative model or by models derived from the individual omics datasets were compared. Interestingly there was very little overlap between gene symbols identified by the individual omics models and the integrative model (**Supplementary Figure 7**). To rule out the possibility that the approaches were simply identifying different, but functionally-redundant, genes (involved in the same biological functions), the feature sets were assessed for pathway over-representation, and it was found that the various models identified largely distinct biological pathways (**Supplementary Figure 7**). Moreover, the features identified by the integrative model were enriched for a larger number of curated gene sets (Broad Institute MSigDB C2 collection), when compared to those identified individually based on data for the individual omics methods, suggesting that the integrative model features were consistent with well annotated biological pathways. We have made similar observations in a number of larger multi-omics studies (14, 17).

Additionally, to assess the biological function of DIABLO selected features (from transcriptomics, proteomics, and epigenomics), we used NetworkAnalyst to construct PPI networks to determine whether these genes and proteins formed an interconnected biological network (**Figure 5B**). Importantly the resultant first-order minimally-connected network was highly integrated and composed of nodes from each of the omics methods, indicating that transcriptomic, proteomic, and epigenomic data were reporting on the same underlying biology. Furthermore, we identified additional nodes from this network that might provide insights into the effectiveness of the vaccine response, including: VDR (vitamin D receptor, involved in T cell function and influences HBV responses) (37); IL18 (pro-inflammatory cytokine for T-helper and NK cells) (38, 39); IKBKE (modulates T cell responses and essential for antiviral responses) (40); and ILF3 (participates in the innate antiviral response) (41); TMEM173 (innate immune signalling) (42); and BCL2L1 (minor role in inflammation attenuation) (43). As previously observed, pathway enrichment using both the statistical integration method DIABLO and biologically-driven PPI integration method NetworkAnalyst separately identified similar functional enrichments, highlighting potential pathways at immune baseline that predict HBV vaccine response (**Figure 5C**; **Supplementary Figures 8, 9**; **Supplementary Tables 13**, **14**).

Plotting integration by DIABLO- or NetworkAnalyst-selected features showed that IRF9 (promotes inflammation and type III interferon signaling) was more highly expressed in non-responders **(** **Figure 5D**). In contrast, NEDD4 (E3 ubiquitin ligase that inhibits inflammatory pathways p38α and TNFα) (44) demonstrated lower expression in non-responders (**Figure 5E**). We observed similar correlation patterns in the baseline proteomics data with T cell activation, and proinflammatory dendritic cell, myeloid cell response, positively and negatively correlated, respectively, with vaccine response. This was further supported by a previous transcriptomic study demonstrating that an enrichment of pro-inflammatory pathways at immune baseline leads to a poor HBV vaccine response (45).

Finally, DIABLO identified a number of taxa from the baseline microbiome data, including *Butyricicoccus* and *Phascolarctobacterium*, which were positively associated with anti-HB antibody titre response (**Supplementary Figure 10A**). Interestingly, these two taxa have both been previously shown to regulate host immune responses. *Butyricicoccus* is a butyrate producer that has been used to modulate immune responses (46–48), while *Phascolarctobacterium* showed evidence of reduced abundance in individuals with an anti-inflammatory signatures (based on low lipopolysaccharide-binding protein and C-reactive protein) (49).

## Discussion

A better understanding of the complex regulatory interplay involved in the immune response to vaccination is a necessary step in the development of precision vaccinology. Leveraging systems biology approaches and collections of high-dimensional molecular immune readouts obtained from clinical cohorts may yield important insights. While these datasets are complex to synthesize and analyze, the complementary information they encode may strengthen biological findings and improve the accuracy of predictive models derived from them. Here we performed extensive molecular profiling of individuals receiving HBV vaccine to investigate vaccine response in adults. To our knowledge, this study constitutes the most comprehensive set of molecular immune readouts on a common set of individuals before and after HBV vaccination. Fifteen healthy HBV-seronegative adults received three doses of HBV vaccine to assess the correlate(s) of protection. We profiled the blood of participants before and after vaccination and defined both temporal changes in the various omics following vaccination, as well as baseline characteristics associated with a robust vaccine response (**Figure 1**).

Using multi-omics integration strategies described herein, we were able to identify significant biological features and pathways from a small sample size of 15 participants. Where possible, we leveraged resampling strategies (leave-one-out cross-validation) to ensure the robustness of our findings, though we acknowledge the limits of doing so in so few samples. While larger studies will be able to provide more robust results, ours was designed to further demonstrate the feasibility of a multi-omics approach to studying vaccine response, even when applied to a relatively small cohort. As omics-based vaccine studies with large numbers of participants are prohibitively expensive to conduct, our integrative multi-omics strategy on a smaller cohort will help ensure these larger studies are conducted in a manner which extracts as much biological meaning as possible. Select findings could then be targeted for further validation in larger cohorts, using cost-effective platforms with more well-defined paths to clinical implementation.

When analyzing patterns of change over time following vaccination, we were able to detect certain differences between pre- and post-vaccination samples (**Figures 2**, **3**). In particular, despite the very substantial impact of variation in underlying genetics, diet, environment and microbiome, transcriptomic analyses of participants 14 days post-vaccination compared to each individual’s baseline pre-vaccination still revealed certain changes in the expression of genes such as ELANE and CAMP, both known to have important roles in the immune responses of many immune cells (50, 51). It is also interesting to note that the greatest differences identified by transcriptomics and epigenomics came from different time points, reinforcing the idea that using multiple omics methods can provide a more complete picture of complex biological phenomena through complementation.

Given that baseline immune profiles are known to predict vaccine responses to many agents (7, 45, 50), we were interested in identifying baseline molecular patterns associated with anti-HBs antibody titre response. When we analyzed each omics dataset separately with respect to vaccine responses, as measured by antibody titre (at Day 120, Day 208), we found only modest differences in the immune baseline between responders and non-responders. However, when this small number of methylation sites, differentially expressed transcripts, and proteins were projected onto PPI networks using NetworkAnalyst integration methods, we uncovered potential biological themes based on the principle of “guilt by association” (52). Specifically, PPI linkages between two nodes imply that there is shared biology (given that PPI are based on curated interactions, involving direct binding, consecutive positions in metabolic pathways, or regulatory interactions), such that PPI networks can be mined for mechanistic information, based not on single gene products but consortia of gene products reflecting pathways and ontologies. Thus, when we used NetworkAnalyst to perform multi-omics integration based on function-related PPI networks, novel nodes and enriched pathways important in HBV vaccine response were identified (**Figure 4**, **Supplementary Figures 8, 9**). The integrated networks shown in **Figure 4** demonstrate the benefit of this approach, since a multitude of novel nodes that provide the “glue” to optimize the network, were identified.

In addition, we applied a multivariate statistical method to carry out multi-omics data integration (DIABLO) and identify baseline features that could predict vaccine response. A particular concern with these methods is overfitting, particularly in studies with a relatively small *n* i.e. few (tens, up to a hundred) biological samples, and a substantially larger *p* i.e. number of molecules or variables (several tens of thousands). This is sometimes referred to as the “**small *n* big *p***” (or p>>>n) problem and can result in poor reproducibility and/or models that fail to generalize well to new data. DIABLO implements a number of strategies to tackle these challenges. First, it reduces the influence of noisy variables by means of dimensionality reduction techniques that summarise and/or identify useful and robust information from the data, leveraging penalisation (lasso) to carry out variable selection (18, 19). Second, it cross-references information across biological spaces by utilizing different types of data and looking for reinforcing biological dynamics, which works as long as these data report on the same basic underlying biological mechanisms.

We used DIABLO to derive a multi-omics model capable of predicting vaccine response from baseline molecular profiles. Critically, this DIABLO model outperformed models of comparable complexity derived from the individual omics analyses, highlighting the utility of multi-omics integration (**Figure 5**). Further, we tested whether the improved performance could be due to a reduced tendency to overfit data as a result of the additional imposed constraints, i.e. enforcing covariance across the omics datasets. We compared the features identified by our integrative model with those identified with single omics approaches, and found these to be almost entirely distinct. Moreover, features selected by DIABLO can be used to construct coherent and highly interconnected protein-protein interaction networks (48). These DIABLO selected features were enriched for a greater number of annotated gene sets, and critically delivered data overlapping with our functional integration approach using NetworkAnalyst (**Figure 5**, and **Supplementary Figures 8, 9**). Taken together, the superior performance in cross-validation and selection of largely distinct sets of features demonstrate that DIABLO was less likely to identify spurious associations and overfit the data on which it was trained. Furthermore, these results suggest that, by taking advantage of the differing effect of background noise and technical confounders across the various omics, and focusing on the consensus information related to the outcome, multi-omics integration can reduce overfitting and result in more robust and generalizable models (14, 17), even in studies where p>>>n.

Functionally, pathway enrichment of our integrative analyses comparing responders and non-responders using NetworkAnalyst and DIABLO yielded some of the same pathways that provide insights into immune baseline features that may contribute to HBV vaccine response (**Figure 5**, and **Supplementary Figures 8, 9**). This overlap in significant biological phenomena reinforces the validity of these two approaches used separately and conjointly since they converged on the same (or similar) biology, including several key innate immune pathways such as JAK-STAT signalling/IL-12 stimulation, TLR activation and neutrophil degranulation. The JAK-STAT signalling pathway is an anti-viral pathway, and modulation of this pathway would play an important role in an effective response to infection, and most likely, vaccination (53). The apparent role of TLR4 signalling is also in agreement with studies showing the important role of this signalling pathway during chronic HBV infection (54) and T cell activation (55). Furthermore, TLR4 signalling cascade as well as IL12 and TLR/IL-1 signaling, are important in the response to vaccines with aluminum-based adjuvants (which is used in ENGERIX^®^-B), suggesting that individuals who respond to vaccination may have greater intrinsic responses to adjuvant compared to non-responders (54, 56). Additionally, pathway enrichment of both integration methods revealed adaptive immune signatures such as IL-17 signalling and Th17 cell differentiation. Lastly, interferon signaling, identified by both integration methods, may play a role in linking innate and adaptive immunity through signal transduction *via* inflammasomes (such as the NLRP3) (57, 58).

In this study, two participants did not reach the minimum titre threshold to be considered protected against HBV infection, even after receiving all three vaccine doses. These individuals were 63 and 72 years of age, placing them in the upper end of the range within our cohort of 15 adults. This implies that immunosenescence (3) may be a contributing factor towards their lack of response to HBV vaccination. To investigate this possibility further, we examined the potential relationship between titre (measured at Day 180) and age acceleration as defined through DNA methylation markers (59–61), and saw no correlation between the two (**Supplementary Figure 4**). It also warrants mention that other participants of equivalent or older age did respond well by the clinical endpoint, indicating that there is likely more than age-related immune changes at play.

One important limitation of our integration paradigm is that, in order to increase statistical power, the information extracted from the data in this manner must be statistically independent, implying a null correlation between sets of identified information. The proposed framework is only attainable if complementary information exists *between* data sets (i.e. flow cytometry, epigenomic, transcriptomic, and proteomic profiling, and fecal 16S rRNA microbiome), and can be extracted with a statistical model that will appropriately aggregate independent information to increase statistical power. Such a requirement may seem to go against the key biological assumption that molecular data are inherently interrelated, i.e. it is believed that they act in unison within biological pathways. However, it remains unclear whether interrelatedness between molecules of different types directly implies statistical correlation (62, 63). The distinct patterns of temporal response across molecular data identified in the current study suggests the underlying biological complexity will be difficult to adequately capture statistically, as e.g. DNA methylation and transcript abundance leading to delayed changes in protein and cell abundances follow different time lines.

In summary, single omics analysis revealed some important signatures and showed trends when contrasting vaccine responder groups. In line with previous studies, our work demonstrated that integrative data analysis across several biological domains can provide a comprehensive view of the molecular pathways and biological networks important in vaccine responses (14, 64, 65). Importantly, our findings revealed that data integration of pre-immunization multi-omics signatures in a small sample size can predict response to HBV vaccination.

## Data Availability Statement

RNA-Seq data was deposited to NCBI’s Gene Expression Omnibus (GEO), and is available under GSE155198. The mass spectrometry proteomics data have been deposited to the ProteomeXchange Consortium via the PRIDE partner repository with the dataset identifier PXD020474. Microbiome data is available at the NCBI Short Read Archive (SRA) under accession number PRJNA658597. Flow cytometry data is available at flowRepository with the ID FR-FCM-Z2R9. Epigenetic data has been submitted to GEO and is available under GSE161020.

## Ethics Statement

The studies involving human participants were reviewed and approved by the University of British Columbia Clinical Research Ethics Board. The patients/participants provided their written informed consent to participate in this study.

## Author Contributions

CS advised on all omics analyses and carried out DIABLO integrative analysis. TB performed RNA-Seq and proteomics bioinformatics analyses. RB-O, TK, and MS recruited patients, collected samples, and coordinated data acquisition. NG and RE performed epigenomic analyses. NA performed microbiome sample processing and analyses. SD performed immune cell phenotyping bioinformatics analysis. QC performed initial screening and processing of proteomics samples. MK performed HBV serology for all participants. LF supervised proteomics sample collection, processing, and analysis. MS supervised epigenomics sample collection, processing, and analysis. WWM supervised microbiome sample collection, processing, and analysis. RB supervised immune cell phenotyping bioinformatics analysis. KL conceived and supervised the DIABLO integrative analysis. RHC supervised data deposition and contributed to conception of the manuscript. ST supervised the DIABLO integrative analysis. RH coordinated RNA-Seq sample collection and processing and supervised the RNA-Seq analysis. WK conceived the study and secured funding. TK and MS conceived the study and secured funding. CS, ST, RH, TK, MS and AL contributed to the conception of the manuscript. AL guided and advised on all omics and integrative analyses. CS, TB, RB-O, NG, NA, SD, LF, WWM, KLC, RH, TK, MS, and AL all contributed to manuscript writing. All authors contributed to the article and approved the submitted version.

## Funding

We acknowledge funding from the Human Vaccines Project. REWH was the recipient of a UBC Killam Professorship and a Canada Research Chair in Health and Genomics. REWH acknowledges funding from the Canadian Institutes of Health Research (CIHR) [funding reference number FDN-154287]. Mass spectrometry infrastructure used here was funded by the Canada Foundation for Innovation and the BC Knowledge Development Fund. Its operation is supported by Genome Canada and Genome BC (214PRO). NA and WWM were supported by a Project Grant (148781) from the Canadian Institutes for Health Research. MS is supported *via* salary awards from the BC Children’s Hospital Foundation, the Canadian Child Health Clinician Scientist Program and the Michael Smith Foundation for Health Research. MS has been an investigator on projects funded by GlaxoSmithKline, Merck, Pfizer, Sanofi-Pasteur, Seqirus, Symvivo and VBI Vaccines. All funds have been paid to his institute, and he has not received any personal payments. KL was supported in part by the National Health and Medical Research Council (NHMRC) Career Development fellowship (GNT1159458). TB, CS, and ST were supported by the National Institute of Health/National Institute of Allergy & Infectious Diseases Human Immunology Project Consortium Grant 5U19AI118608. AL is supported by Simon Fraser University New Faculty Start-up Grant.

## Conflict of Interest

The authors declare that the research was conducted in the absence of any commercial or financial relationships that could be construed as a potential conflict of interest.
