# Supplementary material for: Multi-Omic Data Integration Allows Baseline Immune Signatures to Predict Hepatitis B Vaccine Response in a Small Cohort
Source: Front Immunol. 2020 Nov 30;11:578801. doi: 10.3389/fimmu.2020.578801 (PMC7734088; doi:10.3389/fimmu.2020.578801)
Supplement: Supplementary file 1 [file DataSheet_1.pdf]

SUPPLEMENTARY FIGURES

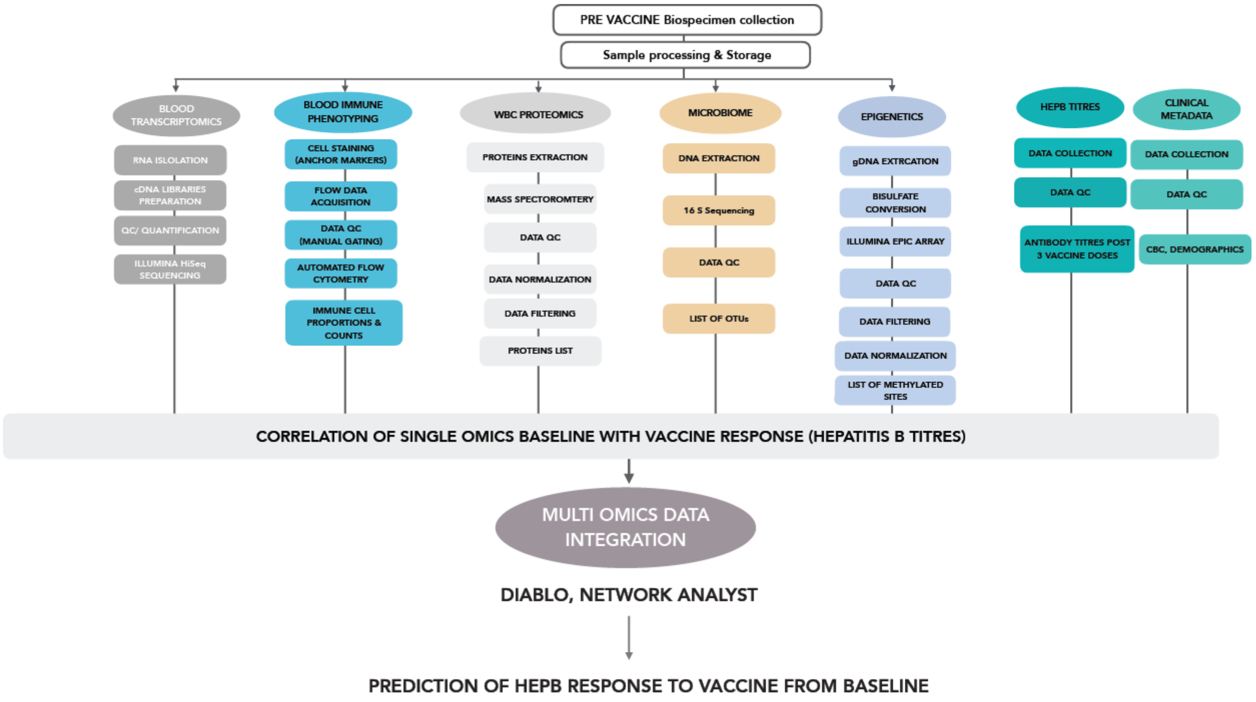

Supplementary Figure 1

Supplementary Figure 1. Work-flow diagram. Sample processing steps and bioinformatics analysis steps are graphically summarized.

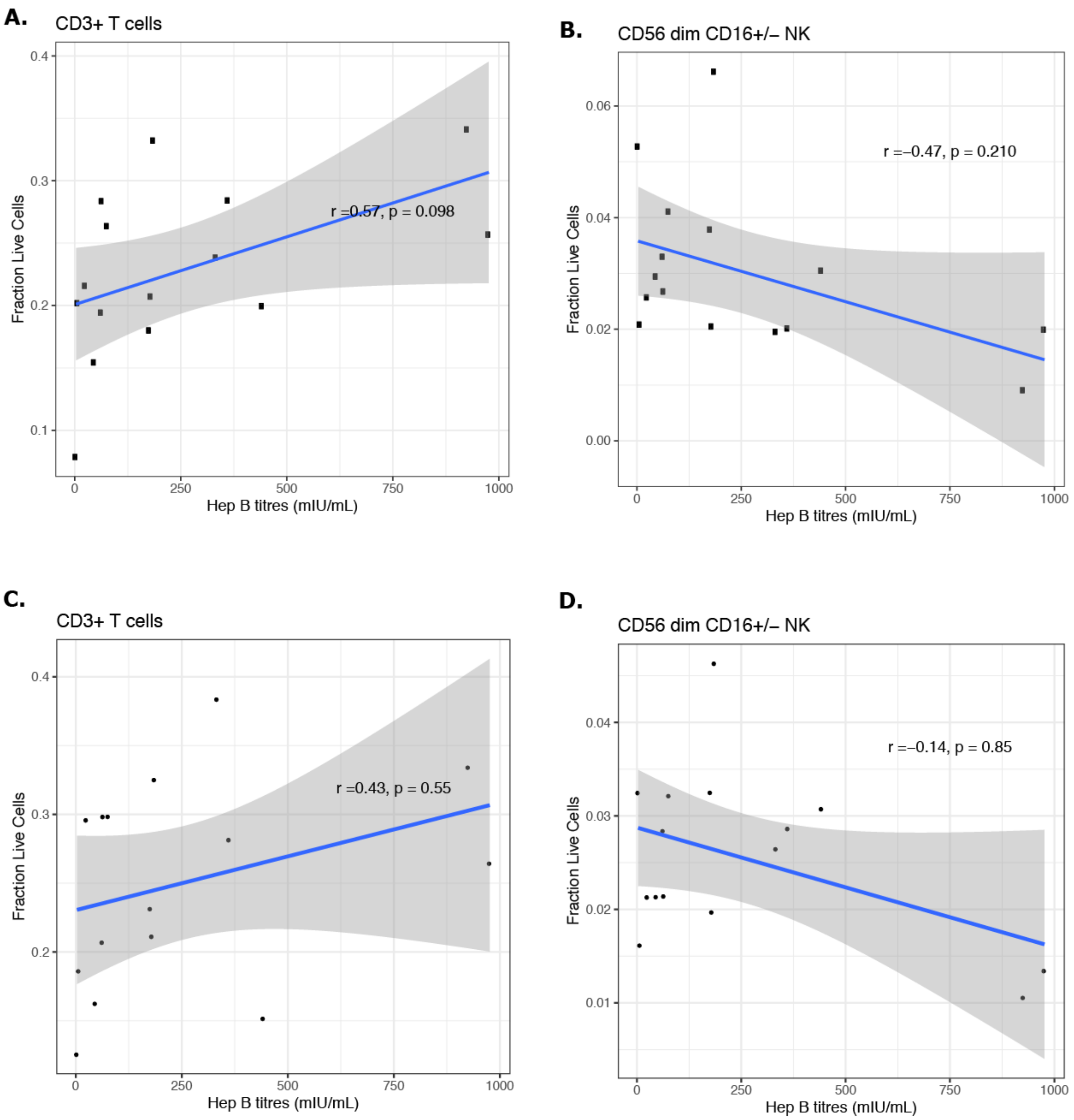

Supplementary Figure 2

Supplementary Figure 2. Spearman correlation of Day 180 HBV antibody titres with abundance of two cell types measured with flow cytometry at Day 7 and 14. A, **C**. CD3+ T cells show a positive correlation with titre levels at Day 7 and Day 14, respectively. **B**, **D**. CD56dim CD56+/- NK cells display a negative correlation with increasing titres at Day 7 and Day 14, respectively.

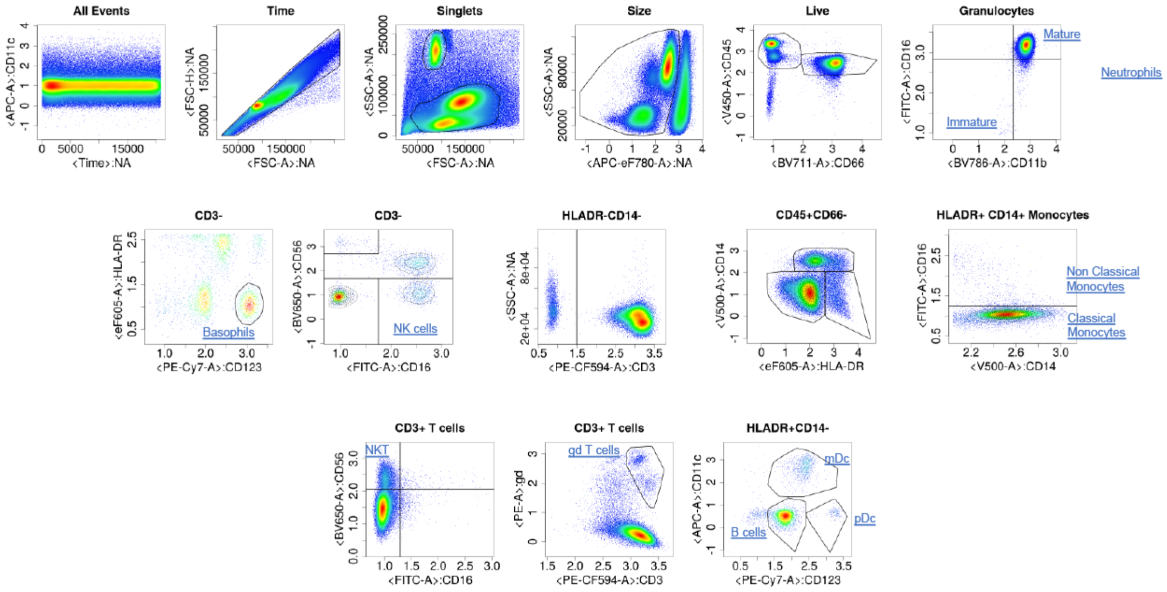

Supplementary Figure 3

Supplementary Figure 3. Gating strategy for immune cell phenotyping. Overview of the gating strategy employed for immune cell phenotyping via flow cytometry.

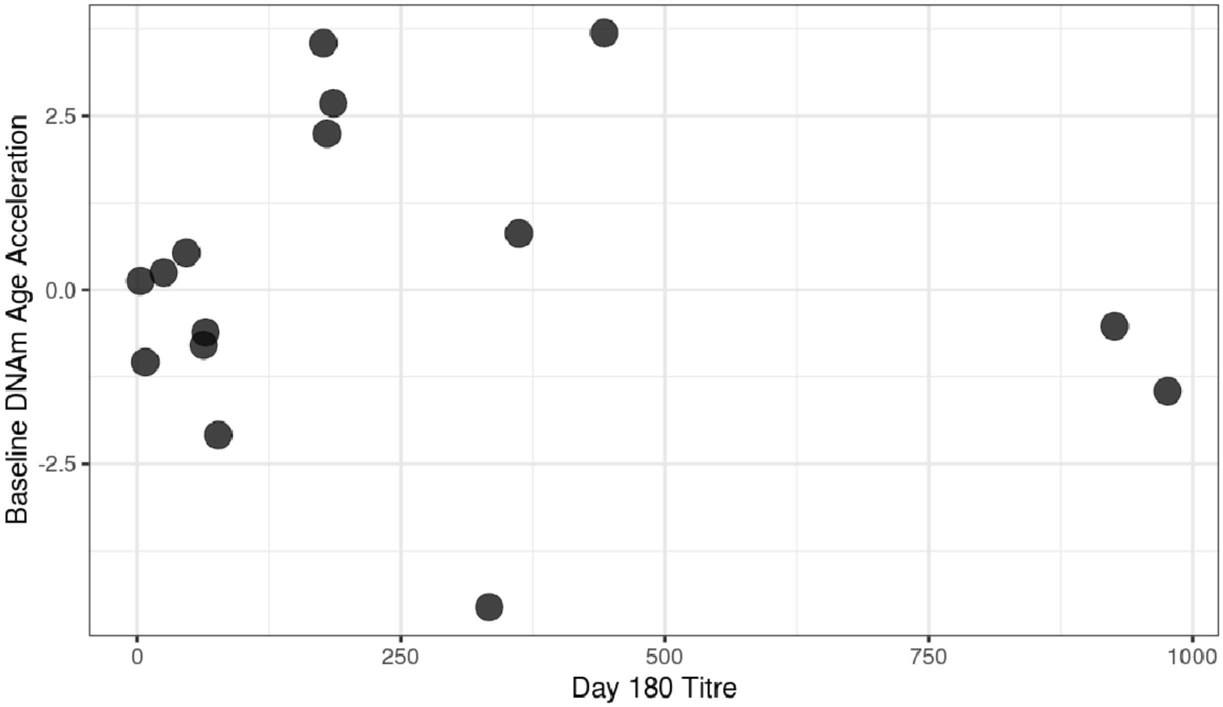

Supplementary Figure 4. Relationship between anti-HBs antibody titres and DNA-methylation (DNAm) age acceleration. No correlation was found between DNAm age acceleration and titres measured at Day 180 (the same time as was used in the various omics analyses).

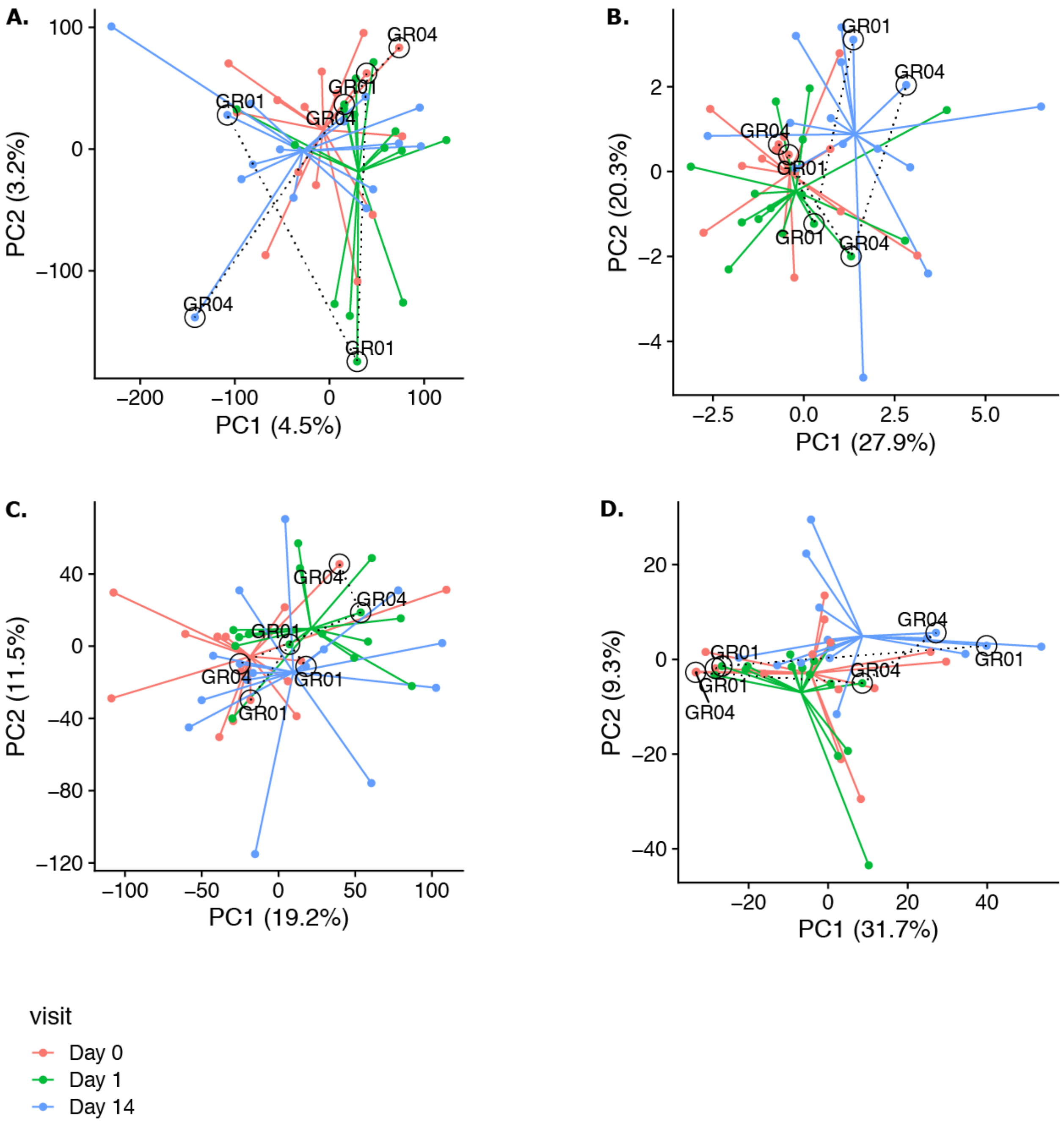

Supplementary Figure 5

Supplementary Figure 5. PCA of various omics analyses, highlighting the two early responders. Panels included here represent **A**. CpG; **B**. flow cytometry; **C**. mRNA; and **D**. proteomics.

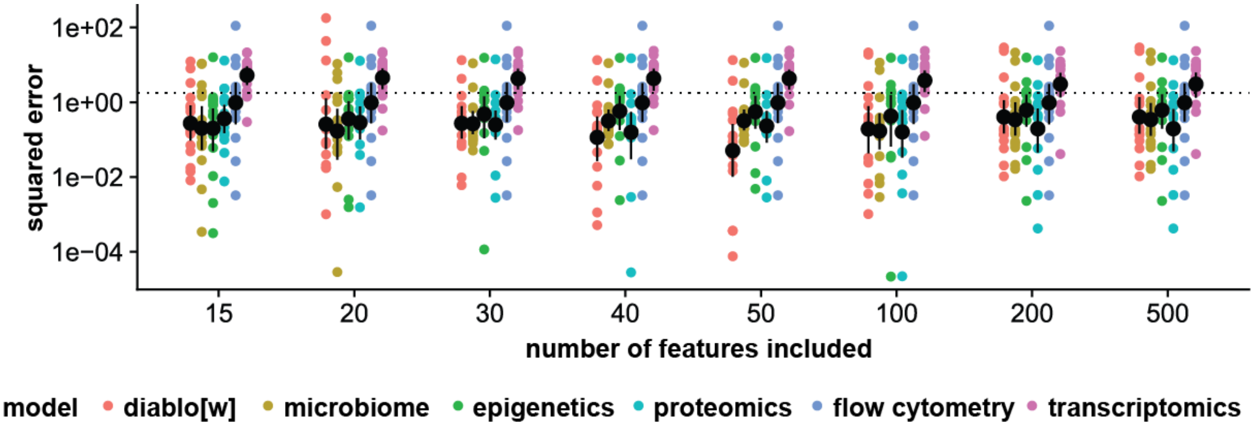

Supplementary Figure 6. Multi-omic integration with DIABLO improves predictive performance. We fit models of increasing complexity (increasing number of features selected) either using DIABLO (**red**) to integrate across the available omic profiles or the individual omics alone using sPLS (**yellow**, microbiome operational taxonomic units; **green**, blood-based DNA methylation; **cyan**, plasma proteomics; **magenta,** whole blood transcriptomics). Performance was evaluated using leave-one-out cross-validation (mean-squared error; lower is better). The best performance was achieved by the DIABLO model retaining 50 features for each omics data type.

A.

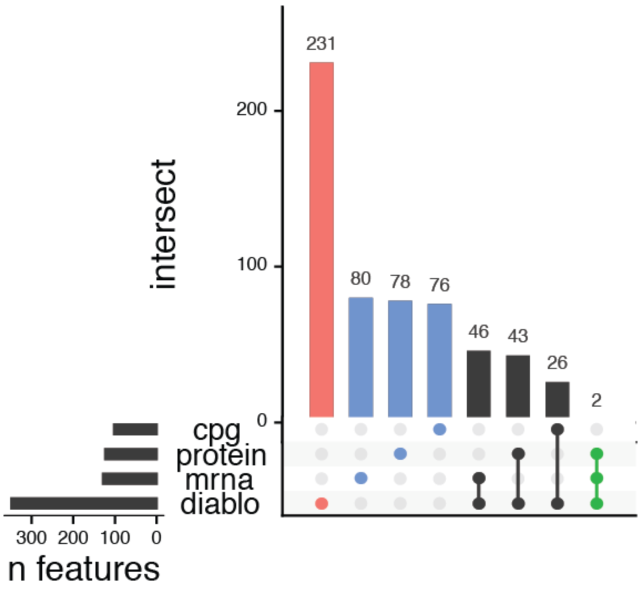

B.

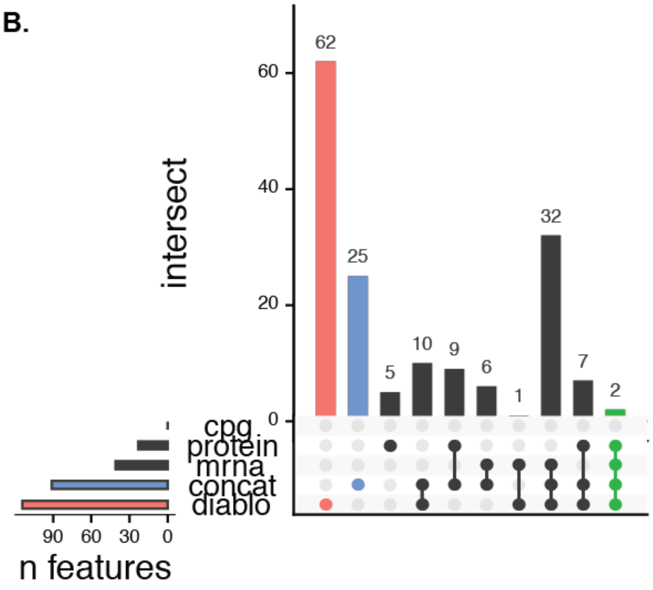

Supplementary Figure 7

Supplementary Figure 7. Multi-omics integration to reduce overfitting by identifying of more biologically relevant features. A. Comparison of the features (mapped to gene symbol as outlined in Materials and Methods - Mapping of Identifiers to Facilitate Biological Interpretation) selected by the best performing multi-omics model (DIABLO; red) to that of single-omics models (sPLS; blue) of equivalent complexity (cpg: blood-based DNA methylation; protein: plasma proteomics; mrna: whole blood transcriptomics).

B. Pathway over-representation analysis using the features described above. Gene sets found to be significantly enriched (adjusted p-value < 0.01) for each set of features were compared.

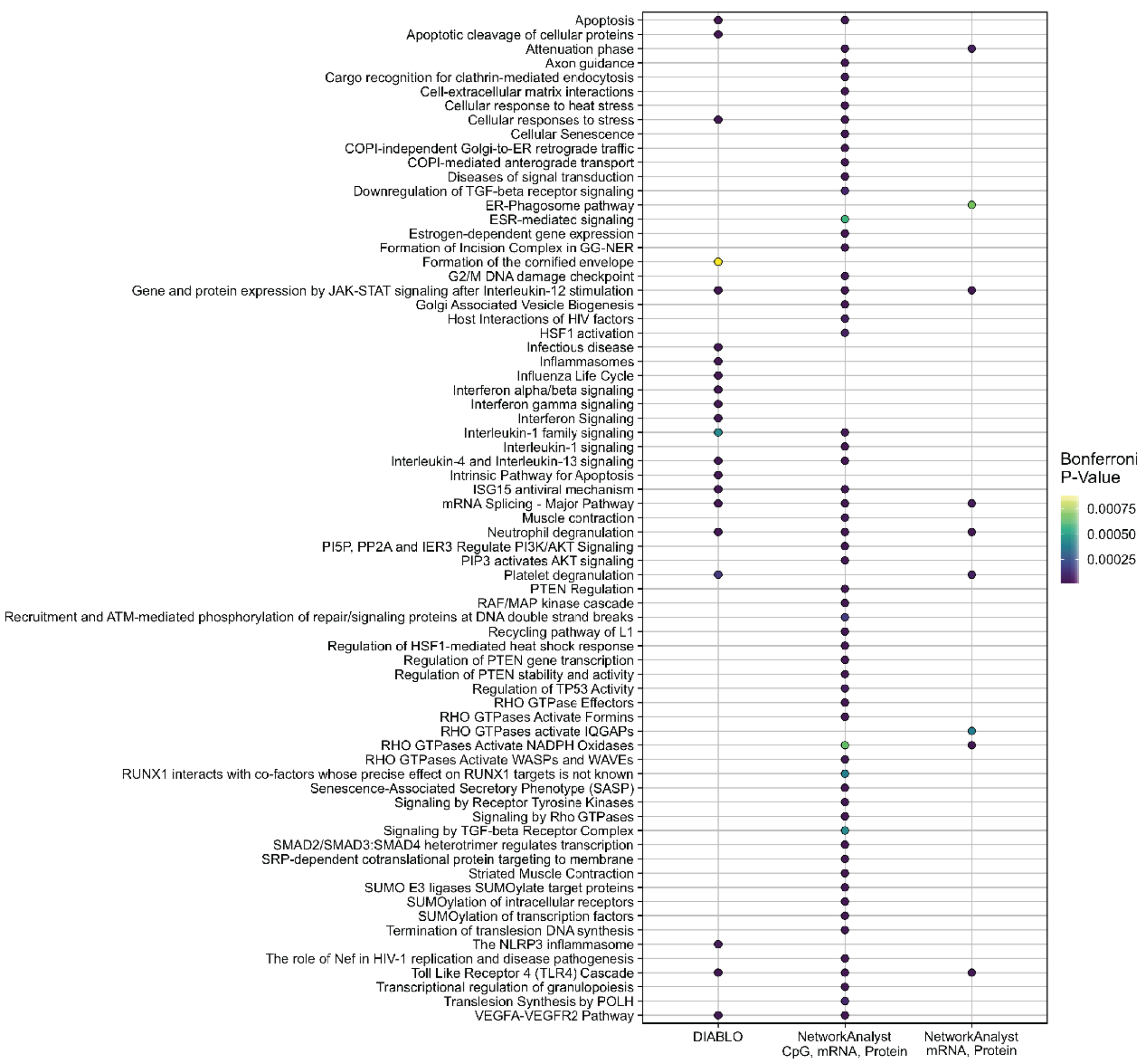

Supplementary Figure 8

Supplementary Figure 8. Dot plot comparing Sigora pathway enrichment results using data from Reactome. Testing was performed on node tables for three networks, in order from left to right: one built from features identified by DIABLO (CpG, mRNA, and Protein); integrated network using CpG, mRNA, and Protein results for responder/titre analyses; and integrated network using mRNA and Protein results for responder vs. non-responder. Results were filtered for significance using Bonferroni-corrected p-value < 0.001.

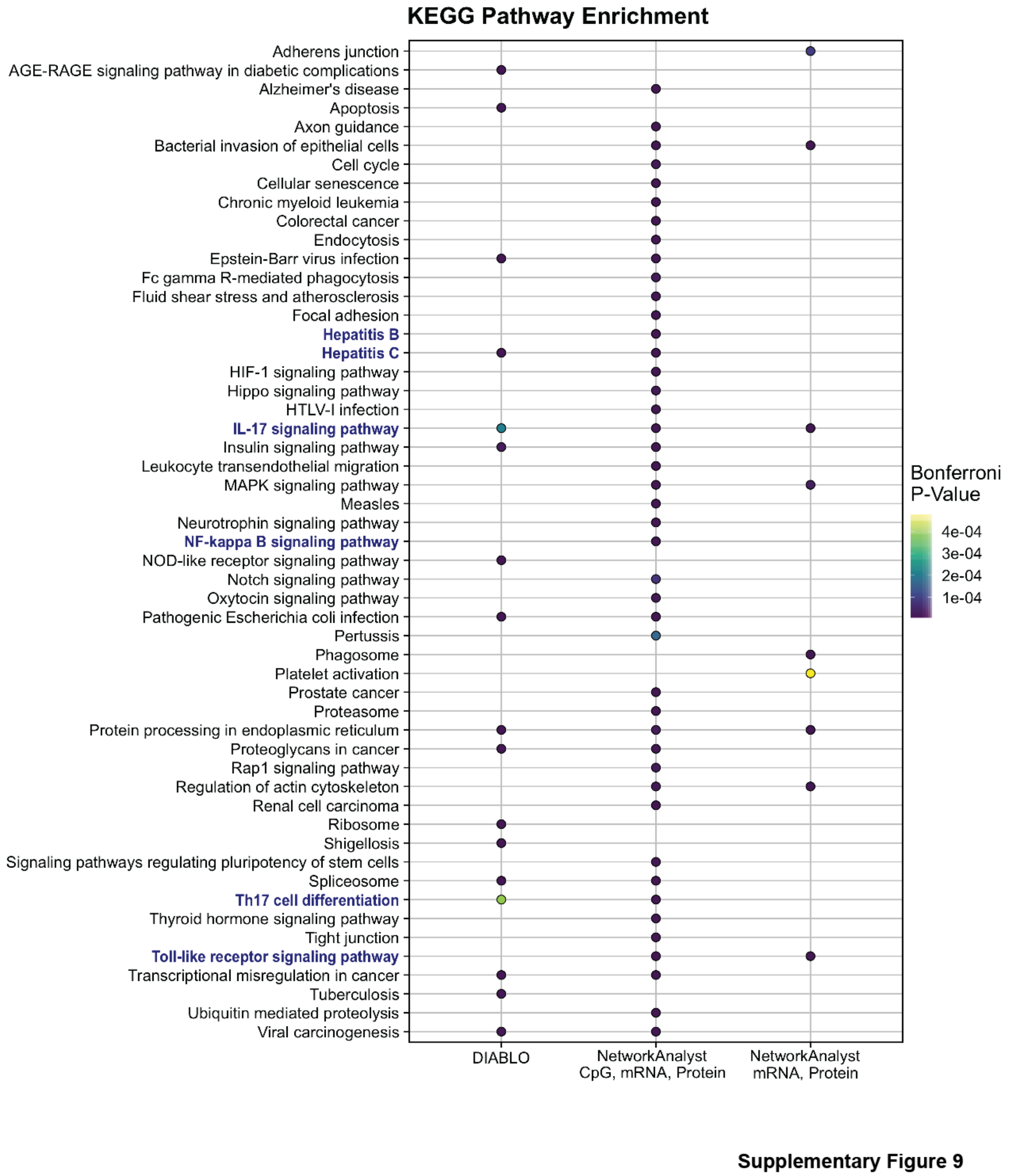

Supplementary Figure 9. Dot plot comparing Sigora pathway enrichment results using data from KEGG. Testing was performed on node tables for three networks, in order from left to right: one built from features identified by DIABLO (CpG, mRNA, and Protein); integrated network using CpG, mRNA, and Protein results for responder/titre analyses; and integrated network using mRNA and Protein results for responder vs. non-responder. Results were filtered for significance using Bonferroni-corrected p-value < 0.001. Highlighted are a few pathways particularly relevant for this analysis.

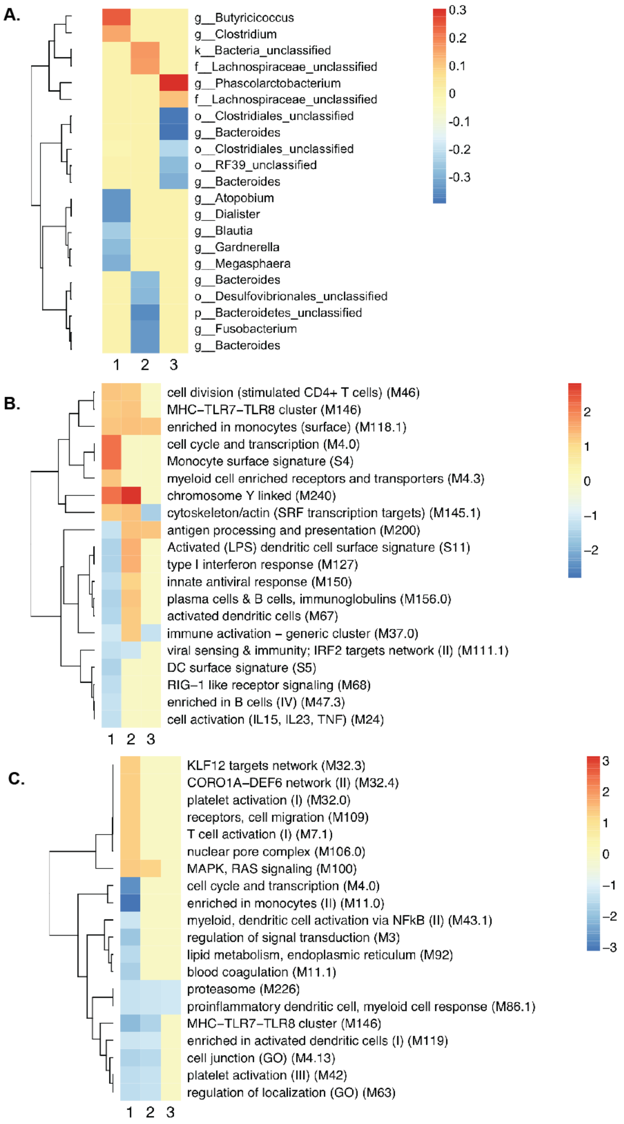

Supplementary Figure 10

Supplementary Figure 10. DIABLO loadings highlight the relationship between baseline immune status and Hepatitis-B vaccine response. Feature loadings for the best performing DIABLO model are visualized using heatmaps (**A**. otu: operational taxonomic units; **B**. mrna: whole blood transcriptomics; **C**. protein: plasma proteomics). For the transcriptomics (**B**) and proteomics (**C**), features were mapped to gene symbol as outlined in Materials and Methods - Mapping of Identifiers to Facilitate Biological Interpretation and we used the coefficient weights of the selected features to carry out gene set enrichment analysis [1] against the Blood Transcriptome Modules [2] collection to facilitate interpretation.

SUPPLEMENTARY FILES

Table_S1_myeloid_panel_markers.xlsx

Table of the 15 antibody markers used in flow cytometry analysis.

Table_S2_CpG_day_analysis.xlsx

Differentially methylated sites identified in epigenetic analysis, comparing each post-vaccination time point to its pre-vaccine counterpart. Results were considered significant with p-value ≤ 0.005 and absolute effect size change of ≥ 5%.

Table_S3_mRNA_day14_vs_day0_DE_genes.xlsx

Differentially expressed genes found when comparing Day 14 (post-vaccination) to Day 0 (pre-vaccination) samples in a paired analysis. Filtered for adjusted p-value < 0.05 and absolute fold change > 1.5.

Table_S4_mRNA_Day180_responder_vs_non_DE_genes.xlsx

Differentially expressed genes found when comparing responders vs. non-responders (defined using Day 180 titres). Filtered for adjusted p-value < 0.05.

Table_S5_proteins_day180_responder_vs_non_DA_proteins.xlsx

Differentially abundant proteins identified from comparing responder and non-responder participants (defined using Day 180 titre measures) using pre-vaccine profiles (samples). Filtered for adjusted p-value < 0.05.

Table_S6_proteins_day180_responder_vs_non_pathways.xlsx

Enriched pathways identified by Sigora when tested on DA proteins for responders vs. non-responders (Day 180 titre measures). Filtered for significance using Bonferroni-corrected p-value ≤ 0.001.

Table_S7_CpG_day180_titre_DM_sites.xlsx

Differentially methylated sites identified in epigenetic analysis, using Day 180 titres as a continuous variable. Results were considered significant with p-value ≤ 0.005 and absolute effect size change of ≥ 5%.

Table_S8_CpG_day180_titre_pathways.xlsx

Enriched pathways found using resources from Reactome.org using the differentially methylated sites correlated with Day 180 titre measures. Results were considered significant with p-value ≤ 0.005.

Table_S9_networkanalyst_CpG_mRNA_proteins_day180_response_pathways.xlsx

Enriched Reactome pathways identified by Sigora using nodes from integrated network of responders vs. non-responders (defined with Day 180 titre values), from epigenetics, transcriptomics, and proteomics results. Filtered for significance using Bonferroni-corrected p-value < 0.001.

Table_S10_networkanalyst_CpG_mRNA_proteins_day180_response_KEGG_pathways.xlsx

Enriched KEGG pathways identified by Sigora using nodes from integrated network of responders vs. non-responders (defined with Day 180 titre values), from epigenetics, transcriptomics, and proteomics results. Filtered for significance using Bonferroni-corrected p-value < 0.001.

Table_S11_networkanalyst_mRNA_proteins_day180_response_pathways.xlsx

Enriched Reactome pathways identified by Sigora using nodes from integrated network of responders vs. non-responders (defined with Day 180 titre values), from transcriptomics and proteomics results. Filtered for significance using Bonferroni-corrected p-value < 0.001.

Table_S12_networkanalyst_mRNA_proteins_day180_response_KEGG_pathways.xlsx

Enriched KEGG pathways identified by Sigora using nodes from integrated network of responders vs. non-responders (defined with Day 180 titre values), from transcriptomics and proteomics results. Filtered for significance using Bonferroni-corrected p-value < 0.001.

Table_S13_diablo_features_network_pathways.xlsx

Reactome pathways found to be enriched by Sigora for node table from NetworkAnalyst combining three feature sets from the output of DIABLO (epigenetics, proteomics, and transcriptomics). Filtered for significance using Bonferroni-corrected p-value < 0.001.

Table_S14_diablo_features_network_KEGG_pathways.xlsx

KEGG pathways found to be enriched by Sigora for node table from NetworkAnalyst combining three feature sets from the output of DIABLO (epigenetics, proteomics, and transcriptomics). Filtered for significance using Bonferroni-corrected p-value < 0.001.

SUPPLEMENTARY MATERIALS AND METHODS

Immune cell phenotyping

Cell fixation was done for two main reasons. First, given that we have longitudinal samples, we wanted the longitudinal samples from the patient as well as the entire cohort to be analyzed together to minimize batch effects. Second, fixing allows us to keep the cells at a particular static state and allows us to make comparison between samples from participants across all cohorts.
After plasma collection, the remaining blood fraction was diluted twice in RPMI and kept on ice, retaining a total of 900uL of the mixture for downstream flow cytometry analysis. Smart tube lysing and fixing solutions were used while follow the manufacturer's recommendations, and as described in Lee et al, 2019 [3]. Briefly, heparinized blood (225uL) was combined with 20mM EDTA (22uL) in cryovials before mixing via gentle pipetting and the addition of 2uL of fixable viability dye (eBioscience catalog # 65-0865-14). This mixture was then incubated in the dark at 4°C, followed by red blood cell lysis via the addition of 350uL of Smart lysis buffer (SmartTube Stable-Lyse V2) and a 15-minute incubation period at room temperature. The stained cells were then fixed with 1mL Smart store buffer (SmartTube Stable-Store V2) for 15 minutes before being transferred to -80°C for storage.
Complete blood counts were undertaken by BC Children's Hospital clinical laboratory to enumerate total white blood cell (WBC) count, plus a differential count including neutrophils, lymphocytes and monocytes using a Sysmex XN-3000 Hematology Analyzer.
For flow cytometry performed on whole blood, fresh samples were stained with fixable viability dye and stored in Smart tube solutions (Smart tube, CA, USA) in -80C. The day of analysis, fixed samples were thawed and cells with a panel of antibodies recognizing anchor markers to identify neutrophils, dendritic cells (DCs), NK cells, monocytes, T cell subsets, basophils and B cells. The full list of 15 markers is included in **Supplementary Table S1**. Flow cytometry data were acquired on a LSRII flow cytometer (BD Biosciences) and analyzed using Flowjo software (version 9.9). Automated gating was undertaken on the same data where a panel of algorithms were used to clean and gate on the cell populations of interest using a combination of markers selected and a set gating strategy. First, Flowcut (https://github.com/jmeskas/flowCut) was used to detect anomalous events that happened during data acquisition. Events were removed if they had the minimum or maximum value in any of the scatter channels, with singlets selected using FSC-A and -W channels. Next, for each sample, flowDensity (a supervised gating algorithm) [4]

was used to determine the thresholds for each marker in each of the biaxial plots in a data-driven manner based on the density distribution of the cells (**Supplementary Figure 3** for gating strategy). Some challenging populations (such as NK subtypes, pDCs and gamma delta T cells) required a second gating step using flowPeaks, an unsupervised clustering algorithm [5]. Finally, cell counts for each cell population identified were obtained and normalized using the bead count, yielding 24 immune cell populations. For additional information, refer to the companion methods paper: *Systems biology methods applied to blood and tissue for a comprehensive analysis of immune response to hepatitis B vaccine in adults*.

Whole blood RNA sequencing

To complete bulk RNA-Seq, RNA was extracted using the PAXGene RNA purification kit (QIAGEN) from whole blood collected in PAXGene tubes. Assessment of RNA quality and quantity was performed using the Agilent 2100 Bioanalyzer (Santa Clara, CA, USA). Poly-adenylated RNA was isolated using the NEBNext Poly(A) mRNA Magnetic Isolation Module (NEB, Ipswich, MA, USA), and cDNA libraries were created using the KAPA Stranded RNA-Seq library preparation kit (Roche, Basel, Switzerland). Samples were sequenced in two batches on the HiSeq2500 with single-end reads of length 100bp. Sequence quality was assessed using FastQC v0.11.7 (https://www.bioinformatics.babraham.ac.uk/projects/fastqc/) and MultiQC v1.0 [6]. Alignment to the reference Ensembl genome GRCh38 v91 [7] was done using STAR v2.5.4b [8] to generate position-sorted BAM files. Counts were generated using the htseq-count function from HTSeq v0.10.0 [9]. Analysis of RNA-Seq data was conducted using R v3.6.3 (https://www.R-project.org/). Count files were filtered to remove globin genes, and any genes with fewer than 10 counts across 15 samples (the number of biological replicates). After filtering and globin removal, samples had a median library size of 4,842,497, with a minimum of 2,217,471 and maximum of 14,755,180 reads. DESeq2 v1.26.0 [10] was used to identify differentially expressed (DE) genes. For comparison of responders and non-responders (defined in the Methods section), sequencing batch was included in the design formula to control any batch effects. DE genes were identified using the Wald statistics test, followed by filtering for significance using a combined threshold of adjusted p-value ≤0.05 and absolute fold-changes ≥1.5. In the case of comparing responders and non-responders (defined using Day 180 titre as previously described), only an adjusted p-value cut-off was used. For pathway enrichment analysis, Sigora v3.0.5 [11] was used with the Reactome database at a hierarchy level of four.

Correction for multiple comparisons was done using the Bonferroni method, filtering results based on a value of ≤ 0.001. For additional information, refer to the companion methods paper: *Systems biology methods applied to blood and tissue for a comprehensive analysis of immune response to hepatitis B vaccine in adults*.

Since blood samples taken for omics analysis were not collected at the same time for all participants, we used an EigenR2 analysis [12] to check the contribution of sampling time to the overall variance for the RNA-Seq data, and found it to be negligible (approximately 2%).

Whole blood epigenomic analysis

200 µL of whole blood freshly collected from the participants were frozen at -80C. Blood samples were thawed and used to extract genomic DNA (gDNA) using DNeasy Blood & Tissue Kit (Qiagen, Hilden, Germany) following manufacturer's instructions. A bisulfate conversion was then performed using an EZ DNA Methylation kit (ZymoResearch, Irvine, CA). A total of 160 ng of bisulfite converted DNA (bcDNA) were used to perform global DNA methylation (DNAm) profiling on the Illumina MethylationEPIC bead chips. Using the Illumina GenomeStudio software package, average beta values were calculated by dividing the methylated probe signal intensity by the sum of methylated and unmethylated probe signal intensities. To give a quantitative measure of relative DNA methylation, average beta values range from 0 (completely unmethylated) to 1 (fully methylated) for each site.

First, the beta value distributions of the DNA methylation (DNAm) data was assessed using the R statistical software. Hierarchical clustering using either the entire EPIC array or 59 SNP probe's beta values was performed using the EPIC array beta values. Quality of the DNAm data was checked by filtering sites according to the following criteria: if probes were interrogated as a SNP, had evidence of cross hybridizing to a region of the genome other than the designed target, had a SNP present at the CpG target or single base extension of the probe (polymorphic probes) [13]. Probes were removed if they had a bead count ≤ 3 in 5% of samples, or had 1% of samples with a bad detection p value ≥ 0.05. The dasen method [14] was used for normalization, and variability associated with differing cell type proportions were corrected by regressing out the beta coefficients from linear regressions of the complete blood counts [15; 16]. Batch effects between Sentrix IDs were observed using Principal Component Analysis (PCA) where ComBat [17] was used to correct for Sentrix ID. Data from technical replicates were also assessed to ensure pre-processing and normalization procedures resulted in more comparable measures

between replicates. 101,864 non-variable CpGs were removed as previously described [18] to reduce multiple test correction and improve computational efficiency. For additional information, refer to the companion methods paper: *Systems biology of blood and tissue for comprehensive analysis of immune response to hepatitis B vaccine in adults*.

We analysed the data by comparing DNAm taken 0, 1, 3, 7, and 14 days post vaccine (DPV). To investigate large patterns of DNAm variation we performed a non-indexed principal component analysis (PCA) comparing PC1 and PC2. Subsequently, in order to take advantage of the longitudinal design of the data, we performed an indexed, multilevel PCA which incorporates the intra-individual variation present in the data, increasing power in the analysis.

We then performed a site-specific analysis using a one-way, paired sample, repeated measures ANOVA, with participant ID used for the within subject variable. Effect sizes were calculated by taking the change in DNAm beta values between a given time point from baseline where the average of this change for each site was calculated. To investigate which DNAm sites at baseline are best correlated with titre response at Day 180, we performed a linear model adjusting for age and sex. Delta betas were calculated by taking the beta coefficient from the model of DNAm and titre response and multiplying it by the 5th and 95th percentile range of titre values. A site was considered significant if it had a p value ≤ 0.005 and an absolute effect size change of $\geq 5\%$. Gene enrichment analyses were performed using the open sourced software from reactome.org, where enrichments were significant if p value ≤ 0.005.

Whole blood proteomic analysis

White blood cells (WBC) were collected from fresh whole blood were stored as dry pellet at -80°C until proteomic analyses were performed. WBC were lysed as previously described [19] then 10μg of WBC lysates and plasma were digested as previously reported [20]. Peptides were desalted, dried then chemically dimethylated with light, medium, and heavy formaldehyde [21]. After labelling, 2μg of peptides were injected into the EasynLC-1000 chromatography system (Thermo) and liquid chromatography and mass spectrometry analysis (LC-MS) were performed. Data analysis was done using MaxQuant (v1.5.5.1) and the human UniProt database (downloaded on 15 July, 2017). Batch correction was performed on log2-transformed data using ComBat from the “sva” R package [22]. A paired t-test was used to test for differentially abundant proteins at each post-vaccination time point (Day 1, 3, 7, & 14) compared to the pre-vaccine time point (Day 0). For comparison of non-responders and responders (defined in the

Methods section), a t-test and multiple-testing correction were applied via a custom script in R. For all tests, proteins were filtered for significance using an adjust p-value of 0.05. For additional information, refer to the companion methods paper: *Systems biology methods applied to blood and tissue for a comprehensive analysis of immune response to hepatitis B vaccine in adults*.

Microbiome analysis

From fecal samples, bacterial community composition was analyzed via high-throughput sequencing of bacterial 16S rRNA amplicons, on the Illumina MiSeq platform using a previously described analysis pipeline [23]. Multivariate analyses via the *mixOmics* R package was used to relate microbiome composition to immune responses.

To identify and remove contaminating sequences, three extraction blanks were included. These were generated by first performing three extraction blanks, then using the eluate from these as a template for PCR amplification, and then sequencing in the same run as the biospecimens.

Two positive controls, consisting of cloned SUP05 DNA, were also included (number of copies = 2*10^6). OTUs present in blanks are either introduced from extraction reagents, PCR reagents, or from neighbouring samples during either the extraction or the PCR amplification process. Contaminating OTUs were identified as being present in at least 50% of blanks, with count geometric mean plus one standard deviation was greater than the samples, reflecting previously described properties of contaminants arising from extraction or PCR reagents[24]. This approach also avoids removing common contaminating OTUs from neighbouring samples either during the extraction or PCR amplification steps, as these are of high relative abundance in the samples but low relative abundance or sporadic presence in the blanks. Contaminating OTUs were removed from the dataset. Samples with total read counts under 1000 after contaminant removal were also removed from further analysis. For additional information, refer to the companion methods paper: *Systems biology methods applied to blood and tissue for a comprehensive analysis of immune response to hepatitis B vaccine in adults*.

DIABLO integration analysis

Data Preparation

Prior to integration by DIABLO, normalized data were further processed. First, for each dataset and feature (cells, CpGs, transcripts, proteins, etc.) in turn, we removed those missing (no

measurable quantity reported) in >25% of samples. Next, for each dataset and feature in turn, missing values were imputed to 1/2 the lowest observed value. Finally, only samples with complete profiles across all datasets were retained for integration. This is a technical limitation of the chosen integration approach.

Model Fitting and Performance Evaluation

Hepatitis-B specific antibody titers, measured 30-days following the final of 3 vaccine doses administered (208 days after the first vaccine dose), were the response. Titers were centered and scaled (mean = 0, sd = 1) prior to use. Models were fit either to individual omic data (using sparse projection to latent structure regression; sPLS) or jointly in an integrative approach (using DIABLO). Model performance was evaluated using leave-one-out cross-validation and the mean squared error metric. We carried out limited exploration of the model hyperparameter spaces (n, the number of components, k, the number of features retained per component) for both sPLS and DIABLO. The estimated out-of-sample error rate was also compared to that of an uninformative model (whereby predictions were obtained by simply sampling with replacement from the responses and a mean squared error computed) in order to assess whether the demonstrated level of performance was useful.

SUPPLEMENTARY REFERENCES

[1] A. Subramanian, P. Tamayo, V.K. Mootha, S. Mukherjee, B.L. Ebert, M.A. Gillette, A. Paulovich, S.L. Pomeroy, T.R. Golub, E.S. Lander, and J.P. Mesirov, Gene set enrichment analysis: a knowledge-based approach for interpreting genome-wide expression profiles. Proc Natl Acad Sci U S A 102 (2005) 15545-50.

[2] D. Chaussabel, and N. Baldwin, Democratizing systems immunology with modular transcriptional repertoire analyses. Nat Rev Immunol 14 (2014) 271-80.

[3] A.H. Lee, C.P. Shannon, N. Amenyogbe, T.B. Bennike, J. Diray-Arce, O.T. Idoko, E.E. Gill, R. Ben-Othman, W.S. Pomat, S.D. van Haren, K.L. Cao, M. Cox, A. Darboe, R. Falsafi, D. Ferrari, D.J. Harbeson, D. He, C. Bing, S.J. Hinshaw, J. Ndure, J. Njie-Jobe, M.A. Pettengill, P.C. Richmond, R. Ford, G. Saleu, G. Masiria, J.P. Matlam, W. Kirarock, E. Roberts, M. Malek, G. Sanchez-Schmitz, A. Singh, A. Angelidou, K.K. Smolen, E. Consortium, R.R. Brinkman, A. Ozonoff, R.E.W. Hancock, A.H.J. van den Biggelaar, H. Steen, S.J. Tebbutt, B. Kampmann, O. Levy, and T.R. Kollmann, Dynamic molecular changes during the first week of human life follow a robust developmental trajectory. Nat Commun 10 (2019) 1092.

[4] M. Malek, M.J. Taghiyar, L. Chong, G. Finak, R. Gottardo, and R.R. Brinkman, flowDensity: reproducing manual gating of flow cytometry data by automated density-based cell population identification. Bioinformatics 31 (2015) 606-7.

[5] Y. Ge, and S.C. Sealfon, flowPeaks: a fast unsupervised clustering for flow cytometry data via K-means and density peak finding. Bioinformatics 28 (2012) 2052-8.

[6] P. Ewels, M. Magnusson, S. Lundin, and M. Kaller, MultiQC: summarize analysis results for multiple tools and samples in a single report. Bioinformatics 32 (2016) 3047-8.

[7] D.R. Zerbino, P. Achuthan, W. Akanni, M.R. Amode, D. Barrell, J. Bhai, K. Billis, C. Cummins, A. Gall, C.G. Giron, L. Gil, L. Gordon, L. Haggerty, E. Haskell, T. Hourlier, O.G. Izuogu, S.H. Janacek, T. Juettemann, J.K. To, M.R. Laird, I. Lavidas, Z. Liu, J.E. Loveland, T. Maurel, W. McLaren, B. Moore, J. Mudge, D.N. Murphy, V. Newman, M. Nuhn, D. Ogeh, C.K. Ong, A. Parker, M. Patricio, H.S. Riat, H. Schuilenburg, D. Sheppard, H. Sparrow, K. Taylor, A. Thormann, A. Vullo, B. Walts, A. Zadissa, A. Frankish, S.E. Hunt, M. Kostadima, N. Langridge, F.J. Martin, M. Muffato, E. Perry, M. Ruffier, D.M. Staines, S.J. Trevanion, B.L. Aken, F. Cunningham, A. Yates, and P. Flicek, Ensembl 2018. Nucleic Acids Res 46 (2018) D754-D761.

[8] A. Dobin, C.A. Davis, F. Schlesinger, J. Drenkow, C. Zaleski, S. Jha, P. Batut, M. Chaisson, and T.R. Gingeras, STAR: ultrafast universal RNA-seq aligner. Bioinformatics 29 (2013) 15-21.

[9] S. Anders, P.T. Pyl, and W. Huber, HTSeq--a Python framework to work with high-throughput sequencing data. Bioinformatics 31 (2015) 166-9.

[10] M.I. Love, W. Huber, and S. Anders, Moderated estimation of fold change and dispersion for RNA-seq data with DESeq2. Genome Biol 15 (2014) 550.

[11] A.B. Foroushani, F.S. Brinkman, and D.J. Lynn, Pathway-GPS and SIGORA: identifying relevant pathways based on the over-representation of their gene-pair signatures. PeerJ 1 (2013) e229.

[12] L.S. Chen, and J.D. Storey, Eigen-R2 for dissecting variation in high-dimensional studies. Bioinformatics 24 (2008) 2260-2.

[13] R. Pidsley, E. Zotenko, T.J. Peters, M.G. Lawrence, G.P. Risbridger, P. Molloy, S. Van Djik, B. Muhlhausler, C. Stirzaker, and S.J. Clark, Critical evaluation of the Illumina MethylationEPIC BeadChip microarray for whole-genome DNA methylation profiling. Genome Biol 17 (2016) 208.
[14] R. Pidsley, Y.W. CC, M. Volta, K. Lunnon, J. Mill, and L.C. Schalkwyk, A data-driven approach to preprocessing Illumina 450K methylation array data. BMC Genomics 14 (2013) 293.
[15] M.J. Jones, S.A. Islam, R.D. Edgar, and M.S. Kobor, Adjusting for Cell Type Composition in DNA Methylation Data Using a Regression-Based Approach. Methods Mol Biol 1589 (2017) 99-106.
[16] E.A. Houseman, W.P. Accomando, D.C. Koestler, B.C. Christensen, C.J. Marsit, H.H. Nelson, J.K. Wiencke, and K.T. Kelsey, DNA methylation arrays as surrogate measures of cell mixture distribution. BMC Bioinformatics 13 (2012) 86.
[17] W.E. Johnson, C. Li, and A. Rabinovic, Adjusting batch effects in microarray expression data using empirical Bayes methods. Biostatistics 8 (2007) 118-27.
[18] R.D. Edgar, M.J. Jones, M.J. Meaney, G. Turecki, and M.S. Kobor, BECon: a tool for interpreting DNA methylation findings from blood in the context of brain. Transl Psychiatry 7 (2017) e1187.
[19] S. Beck, A. Michalski, O. Raether, M. Lubeck, S. Kaspar, N. Goedecke, C. Baessmann, D. Hornburg, F. Meier, I. Paron, N.A. Kulak, J. Cox, and M. Mann, The Impact II, a Very High-Resolution Quadrupole Time-of-Flight Instrument (QTOF) for Deep Shotgun Proteomics. Mol Cell Proteomics 14 (2015) 2014-29.
[20] Q.W. Chan, A.P. Melathopoulos, S.F. Pernal, and L.J. Foster, The innate immune and systemic response in honey bees to a bacterial pathogen, Paenibacillus larvae. BMC Genomics 10 (2009) 387.
[21] P.J. Boersema, T.T. Aye, T.A. van Veen, A.J. Heck, and S. Mohammed, Triplex protein quantification based on stable isotope labeling by peptide dimethylation applied to cell and tissue lysates. Proteomics 8 (2008) 4624-32.
[22] J.T. Leek, W.E. Johnson, H.S. Parker, A.E. Jaffe, and J.D. Storey, The sva package for removing batch effects and other unwanted variation in high-throughput experiments. Bioinformatics 28 (2012) 882-3.
[23] L.T. Stiemsma, M.C. Arrieta, P.A. Dimitriu, J. Cheng, L. Thorson, D.L. Lefebvre, M.B. Azad, P. Subbarao, P. Mandhane, A. Becker, M.R. Sears, T.R. Kollmann, I. Canadian Healthy Infant Longitudinal Development Study, W.W. Mohn, B.B. Finlay, and S.E. Turvey, Shifts in Lachnospira and Clostridium sp. in the 3-month stool microbiome are associated with preschool age asthma. Clin Sci (Lond) 130 (2016) 2199-2207.
[24] N.M. Davis, D.M. Proctor, S.P. Holmes, D.A. Relman, and B.J. Callahan, Simple statistical identification and removal of contaminant sequences in marker-gene and metagenomics data.
